# Covert-attention shifting superseded: Simple visual search explained by a computational cognitive architecture with early vision limitations, eye movements, and task strategies

**DOI:** 10.3758/s13423-025-02772-9

**Published:** 2026-02-17

**Authors:** David E. Kieras, David E. Meyer

**Affiliations:** 1 University of Michigan, 2560 Bunker Hill Rd, Ann Arbor, MI 48105 USA; 2 Department of Psychology, 2272 E. Hall, University of Michigan, Ann Arbor, MI 48109 USA

**Keywords:** Visual search, Covert attention, Early-vision, Eye movements, Task strategy, Computational models, Cognitive architecture, Individual differences

## Abstract

This article concerns simple visual-search tasks that require people to respond “yes” or “no” about whether a specified target object is present in stimulus displays containing relatively small numbers of typically simple objects. The currently most popular cognitive theories regarding human performance in these tasks claim that a person’s response time depends on the number of shifts of covert visual attention required to choose the response. Such theories provide no significant roles for cognitive task strategies, eye movements, and early-vision limitations (e.g., lower visual resolution and increased crowding effects for displayed objects with greater retinal eccentricity). In contrast, the present research used the EPIC computational cognitive architecture to construct precise simulation models that rely on these more basic mechanisms without assuming any role for covert attention. Results from the simulations show that models systematically incorporating early-vision limitations, eye movements, and parsimonious cognitive task strategies may suffice to account precisely for both the speed and accuracy of human performance during simple visual search. These models succeed at fitting not only empirical data aggregated across participants but also data from different subsets of individual participants who had similar visual parameter values and task strategies. Thus, it appears that covert-attention shifting is not necessary to explain simple visual search. Future models of visual search can be made more veridical and complete by avoiding ill-defined concepts of attention and instead further developing theories of visual mechanisms, task strategies, and motor mechanisms to explain empirical phenomena.

## Visual search

Most people perform visual searches many times daily. For example, various real-world tasks with computers—ranging from familiar activities on personal computers to specialized tasks in military systems—require a person to find objects such as particular icons on a display. In most laboratory visual-search experiments, participants are presented with displays of objects and a *search task* that specifies the *target(s)* for the search (i.e., the to-be-sought object or objects). Other displayed objects are defined by the search-task specification as *distractors*. Participants must respond depending on whether and/or where targets are present in the display.

### Attention in simple visual-search tasks

Laboratory visual-search experiments have varied widely in their specific tasks, stimuli, and procedures (e.g., Buetti et al., 2016; Bundesen & Pedersen, 1983; Duncan & Humphreys, 1989; Najemnik & Geisler, 2005; Williams, 1966; Treisman & Gelade, 1980; Wolfe et al., 1989). However, the present article’s purpose is not to survey the entire field of such research. It is, instead, to focus on a subset of those experiments whose goal has been to reveal the operation of attention in visual perception. There the displays have contained variable numbers of simple objects in relatively large visual fields, and the participants’ task has been to respond about whether a designated target is *present* or *absent* while a display remains visible. We call this a *simple visual-search task.*

Treisman and Gelade (1980) used results from simple visual-search tasks to infer a specific role of attention in the perceptual process. According to their feature-integration theory, attention is *covertly shifted* from one spatial location to another in order to combine separate visual features into integrated representations of visual objects. The present/absent response is chosen after this serial feature-integration process has been completed, and the response time depends on the number of covert attention shifts performed before the response is made.

The research inspired by Treisman and Gelade’s (1980) experimental paradigm and their theory of covert-attention shifting is arguably the largest and most elaborated body of experiments on simple visual search. Subsequent progress based on these seminal contributions has come partly through the efforts of Wolfe and his coworkers. Their work, starting with Wolfe et al. (1989), has further developed theories of covert-attention shifting.

Our goal here is to better understand whether covert-attention shifting plays any significant role in the performance of simple visual-search tasks. We do so by constructing and testing computational models based on the Executive Process Interactive Control (EPIC) cognitive architecture (Kieras & Meyer, 1997; Meyer & Kieras, 1997a, 1999). The following sections focus on the basic paradigm of simple visual search and some representative empirical results from it.

### The simple visual-search paradigm and representative results

To explicitly illustrate the simple visual-search paradigm, we consider a study by Wolfe et al. (2010). Their study is an excellent exemplar, and the publicly available data from it (Visual Attention Lab, n.d.) are of very high quality. Accordingly, they were chosen to be used in subsequent sections of the present article for testing various EPIC computational models of simple visual search.

#### Displays and tasks

**Fig. 1:**
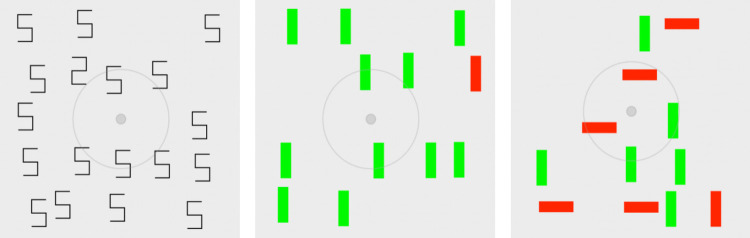
Example displays produced by the model simulations on positive trials in each task condition of Wolfe et al. (2010) for display set size 18. From left to right, the tasks are Shape, Color, and Conjunction. Concentric gray circles indicate the simulated eye location on the display, initially at the designated fixation point. Outer circle: 10° diameter, Inner circle: 1° diameter. (Color figure online)

Figure 1 shows illustrative displays for three simple visual-search tasks studied by Wolfe et al. (2010). For displays like the left-most panel of Fig. 1, participants had to respond with one or the other of two key presses that indicated whether a “digital *2*” shape (the target) was present or absent in a field of “digital *5*” shapes (distractors); we call this the *Shape* task. For displays like the center panel of Fig. 1, participants had to respond whether a red vertical bar was present or absent in a field of green vertical bars; we call this the *Color* task. For displays like the right panel of Fig. 1, participants had to respond whether a red vertical bar was present or absent in a field of red horizontal bars and green vertical bars; we call this the *Conjunction* task (i.e., the target is defined as a conjunction of red and vertical features).

On each trial of each task, the stimulus display contained either an array of distractor objects with a single target object embedded in it (constituting a *positive* trial), or an array of only distractors (constituting a *negative* trial). Across trials, the *set size* (i.e., total number of objects in the display) varied from three to 18. The target was present on half the trials and absent on the other half. The displayed objects appeared at nonoverlapping random locations within a constant-size spatial area. The dependent variables included the response time (RT)—measured from the onset of each display until a key press occurred—and error rate (ER). These variables were analyzed as a function of task type, trial polarity (positive or negative), and display set size.

#### Examples of results from simple visual search

**Fig. 2:**
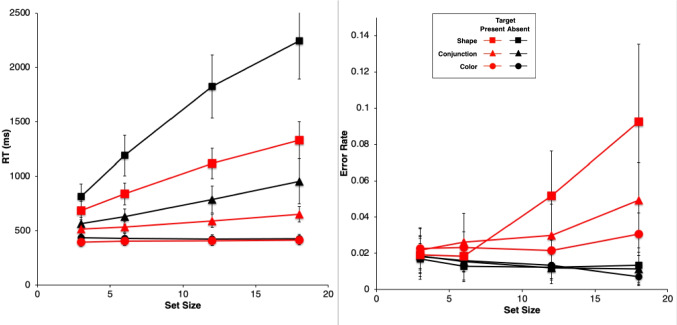
Observed mean RTs (left panel, scale 0–2,500 ms) and ERs (right panel, scale 0–0.15) for each task in the study by Wolfe et al. (2010). Shape: squares, Conjunction: triangles, Color: circles. Negative (target-absent) trials are plotted in black, positive (target-present) trials in red. The error bars are 95% confidence intervals based on standard errors of the individual participants’ means underlying each plotted mean, and they thus reflect between-participant variability. (Color figure online)

Figure 2 shows results from the study by Wolfe et al. (2010). They are very typical data for simple visual-search experiments.

Overall, there is an approximately linear increase in the mean RTs of correct responses as display set size increases (Fig. 2, left panel). The search tasks have RT slopes that range from essentially zero for the Color task to 50 ms/object or greater for the apparently more difficult search tasks, with the Shape task yielding the largest slopes, and the Conjunction task yielding intermediate slopes. When reliably nonzero, the RT slopes for negative trials are roughly twice those for corresponding positive trials, suggesting that a serial self-terminating search process might be involved.

Concomitantly, the ERs also manifest some important effects (Fig. 2, right panel). *Miss* errors (responding “absent” on positive trials) increase with display set size and apparent task difficulty; thus, they are positively correlated with mean RTs. *False-alarm* errors (responding “present” on negative trials) rarely occur; display set size and task difficulty do not appear to affect them much, if at all.

The main factor effects found by Wolfe et al. (2010) first appeared in the seminal studies of simple visual search by Treisman and colleagues (Treisman & Gelade, 1980; Treisman et al., 1977). Specifically, Treisman and Gelade (1980) reported that mean RTs for *single-feature* tasks like the Color task were relatively short and constant as a function of display set size, whereas mean RTs increased linearly with set size for tasks like the Conjunction task, which require processing combinations of features.

Furthermore, Wolfe et al. (1989) found that tasks with apparently difficult discrimination requirements, as in the Shape task, sometimes yielded much steeper RT slopes than did typical Conjunction-like tasks. In fact, the mean RT slopes for Conjunction-like tasks may be relatively shallow, depending on the specific visual characteristics of their stimuli (Wolfe et al., 1989). Thus, many subsequent studies of simple visual search sought to identify what factors govern the RT slopes as a function of display set size, type of simple visual-search task, and the visual features involved.

Other investigators of visual search have postulated additional forms of visual and attentional processing instead of simple covert-attention shifting. They have studied them by using different tasks and stimulus manipulations (e.g., Buetti et al., 2016; Bundesen, 1990; Duncan & Humphreys, 1989; Lleras et al., 2022). Their theories, and the empirical results that inspired them, will be considered further in the General Discussion at the end of this article.

Meanwhile, in the next section, the key assumptions of covert-attention shifting theories for simple visual search are presented and critiqued.

## Hypothetical covert-attention shifting in simple visual search

### Shifts of covert attention instead of overt eye movements

One conceivable explanation for linear mean RTs in simple search tasks (e.g., Fig. 2, left panel) is that participants move their eyes overtly to fixate on each displayed object while performing the search, as has been found sometimes in other types of visual-search experiments. However, typical RT slopes in simple visual search are much less than would be possible if eye movements fixated on each displayed object separately. More specifically, a common rule-of-thumb estimate for eye-fixation durations is 250 ms per fixation, but RT slopes in simple visual search usually range from essentially 0 to about 90 ms per displayed object.

Findings like these have led many past researchers to reject overt eye movements for explaining how simple visual search is performed (cf. Treisman & Gelade, 1980; Wolfe, 2007, pp. 106–107, 2021; Wolfe et al., 1989, 2010). Furthermore, Wolfe et al. (2010) apparently assumed that eye movements would not be involved because their participants were instructed to keep their eyes focused on a central fixation point in the display during each test trial. They (Wolfe et al., 2010) also claimed that the stimuli in their study could “be easily identified outside of the fovea” (p. 1305), which would make eye movements unnecessary.

Taken together, such considerations have been used to justify the belief that covert-attention shifting underlies simple visual-search performance and is much faster than overt eye movements. Following Treisman and Gelade (1980), various versions of sequential covert-attention shifting have been introduced and elaborated by Wolfe in his *Guided Search* model (e.g., Wolfe, 1994, 2007, 2021; Wolfe et al. 1989). Puzzlingly, despite some early contrary evidence (e.g., Pashler, 1987), this line of theory development gives little or no role to eye movements and *early-vision limitations* such as lower acuity and higher crowding with greater eccentricity. Instead, covert-attention shifting theories assume that all visual features of all visible objects are present in the relevant object representations. We term this the *perfect early-vision assumption*.

### Are early-vision limitations and eye movements actually irrelevant?

As is clear in Treisman (1988), the mainstream of visual search theory followed Neisser (1963, 1967) by discounting any role of early-vision limitations and eye movements in favor of his concept of covert focal attention. Nevertheless, as demonstrated in the next two subsections, consideration of early-vision limitations and eye movements is required for coherent and accurate accounts of simple as well as complex visual search.

#### Unjustified dismissal of early-vision limitations

The studies of simple visual search have involved a remarkable dismissal of effects due to retinal inhomogeneity and visual crowding, even though such early-vision limitations have been traditionally well documented in experimental psychology (e.g., Woodworth, 1938; Woodworth & Schlosberg, 1954; cf. Findlay & Gilchrist, 2003; Rosenholtz, 2016). This dismissal has occurred even though some experiments on simple visual search clearly demonstrate the relevance of early-vision limitations (e.g., Carrasco et al., 1995; Carrasco & Frieder, 1996; Wertheim et al., 2006).

##### Attention does not overcome early-vision limitations

The tasks that are typically used for measuring early-vision limitations already incorporate whatever contributions covert attention might make to perception. For example, participants may be instructed to attend to and report about a designated object in the visual field while remaining fixated on a central point. Participants must try to identify the target (e.g., Anstis, 1974) or report its properties when surrounded by flanking objects (e.g., Põder & Wagemans, 2007). Any difficulties in recognizing the target must be due to early-vision limitations rather than an attention-allocation problem (cf. Pelli & Tillman, 2008b). Consequently, *early-vision limitations must influence the difficulty of simple visual search at least as much as they do in psychophysical recognition tasks*.

#### Unjustified dismissal of a role for eye movements

Mainstream theories of covert-attention shifting have also assumed that eye movements are irrelevant in simple visual search. One justification for this dismissal is, as mentioned before, a belief that the target objects are easily identified outside the fovea. If so, then no eye movements would be needed to help identify them. A second justification is that in most experiments on simple visual search, participants have been instructed to maintain fixation on a central point during each trial. A third justification is that, in some experiments, the same effects on search RTs have been obtained regardless of whether eye movements were made or allowed (Wolfe, 2007, pp. 106–107). However, a survey of such experiments shows that differences in ERs, low statistical power, and weak stimulus manipulations render this conclusion dubious.

To the contrary, if eye movements are recorded in simple visual-search tasks, a more typical result is that RTs across trials correlate strongly with the numbers of eye movements made during the trials (e.g., Hulleman & Olivers, 2017; Zelinsky & Sheinberg, 1995, 1997). How many eye movements occur during task trials seems to depend on how many eye fixations are needed to perceive the objects *well enough for completing the task*. This in turn depends on the objects’ early-vision characteristics (such as retinal eccentricity and visual crowding) as well as on the task’s requirements.

More specifically, if objects can be perceived outside the fovea to some extent, then multiple objects can be processed in a fixation. As a result, fewer eye movements will be needed, and the slope of RTs with increasing display set size will be shallower. Consequently, the argument offered by proponents of covert-attention shifting—that RT slopes of simple visual searches are too shallow to be consistent with the occurrence of eye movements—fails to hold (cf. Findlay & Gilchrist, 2003; Hulleman & Olivers, 2017).

## Current status of covert-attention shifting in research on simple visual search

As argued here, theories of covert-attention shifting in simple visual search are seriously flawed because they disregard fundamental mechanisms of the human visual system. Yet the prevailing belief that covert-attention shifting is the primary psychological mechanism for simple visual search continues to dominate. For example, this is evident from recent special issues of *Attention, Perception, & Psychophysics* in honor of Anne Treisman (Wolfe, 2020a, b) and from Wolfe’s (2021) recent update to his Guided Search model. The present situation has persisted even though fundamental problems with the scientific definition of “attention” have been highlighted repeatedly throughout the past several decades (e.g., Allport, 1987, 1993; Anderson, 2011; Hommel et al., 2019; Luck & Vecera, 2002).

In contrast, the present article shows that both RT and ER data from simple visual-search experiments can be precisely accounted for by formal models without any mechanism of covert-attention shifting. These models avoid the scientific opacity of the attention concept and provide explanations that are more parsimonious. Hulleman and Olivers (2017) have introduced similar ideas, starting with the assumption that more than one visual object can be processed in an eye fixation. They showed that the time required for eye movements controlled by a task strategy provides a superior account of simple visual search. The models presented here are similar in spirit to theirs, but explain additional search task situations, while being more detailed and accurate.

The next section introduces our alternative approach, in which early-vision limitations, eye movements, and task strategies are the primary components of computational models for simple visual search.

## Toward a more complete and veridical theory of simple visual search

We propose models of simple visual search constructed by using the EPIC computational cognitive architecture (cf. Kieras & Meyer, 1997; Meyer & Kieras, 1997a, 1999).

### Basic EPIC mechanisms

The models to be presented rely on three well-documented basic mechanisms:

#### Early-vision limitations

Contrary to the perfect early-vision assumption, we firstly assume that early-vision limitations play a crucial role in visual search processes. Human perception of visual features is indubitably limited. Because of retinal inhomogeneity, visual resolving power is highest in the fovea, and crowding effects are more extreme at greater retinal eccentricities.

Moreover, various visual features—here termed visual *properties*^1^*—*differ in how well they can be detected in central versus peripheral vision. Again, this is because of different resolution requirements and susceptibility to crowding. For example, color can often be detected quite well in peripheral vision. However, detailed shape, as in normal-sized letters, often requires foveal vision for recognition, especially if the target letter is flanked by other letters.

#### Eye movements

Secondly, we assume that simple visual search often depends crucially on eye movements. They are essential for bringing objects of interest into the high-resolution and low-crowding areas of the retina. At the same time, information from peripheral vision is not ignored; it might suffice to complete the task without additional eye movements, or it might provide guidance about where the eyes should be moved next. Thus, in a search task, eye movements will be governed by both the visual situation and task requirements.

#### Task strategies

Thirdly, we assume that simple visual search always depends on a cognitive task strategy. To perform a visual search task, participants must acquire and apply task-specific strategies for two major purposes: moving the eyes as needed to collect relevant visual information and making the appropriate response when that information has become available.

The models in this paper use EPIC’s cognitive mechanism for representing and executing task strategies in the form of *production rules*, an explicit and modular representation of procedural knowledge. A specific strategy so represented and executed is a type of mechanism. However, it can be changed by instructions, task demands, and practice, whereas the mechanisms underlying perception and motor movements are presumed to be relatively fixed.

Examples of how explicit task strategies are important for computational cognitive models appear in the original work that developed the EPIC architecture (Kieras & Meyer, 2000; Meyer & Kieras, 1997a, b; Schumacher et al., 2001). There the modeling showed that empirical phenomena conventionally attributed to cognitive constraints such as a “response selection bottleneck” are more veridically explained by cognitive strategies that meet task demands while operating with limited perceptual and motor mechanisms. Similarly, the models in the present article demonstrate that a “covert attention bottleneck” for explaining simple visual search should likewise be replaced by cognitive strategies operating with limited perceptual and motor mechanisms.

#### Modeling visual search with the EPIC cognitive architecture

Previous computational models using the EPIC cognitive architecture account well for data from eye-tracking experiments on visual search tasks involving large numbers of complex objects or computer interface menus (Halverson & Hornof, 2011; Hornof & Halverson, 2003; Hornof & Kieras, 1997, 1999; Kieras, 2010, 2016; Kieras & Hornof, 2014, 2017; Kieras, et al., 2015; Kieras & Marshall, 2006). The present article introduces EPIC models for simple visual search that are closely related to these prior ones, but with a major difference involving the task strategies.

### Overview of subsequent sections

We next cover all the topics related to how our EPIC models work for simple visual search. Then we present further details about the data to be modeled, followed by how the models account for these data in detail. The final part of the article summarizes our conclusions and their implications for future research.

## The EPIC cognitive architecture

### Components and overall operation of EPIC

**Fig. 3:**
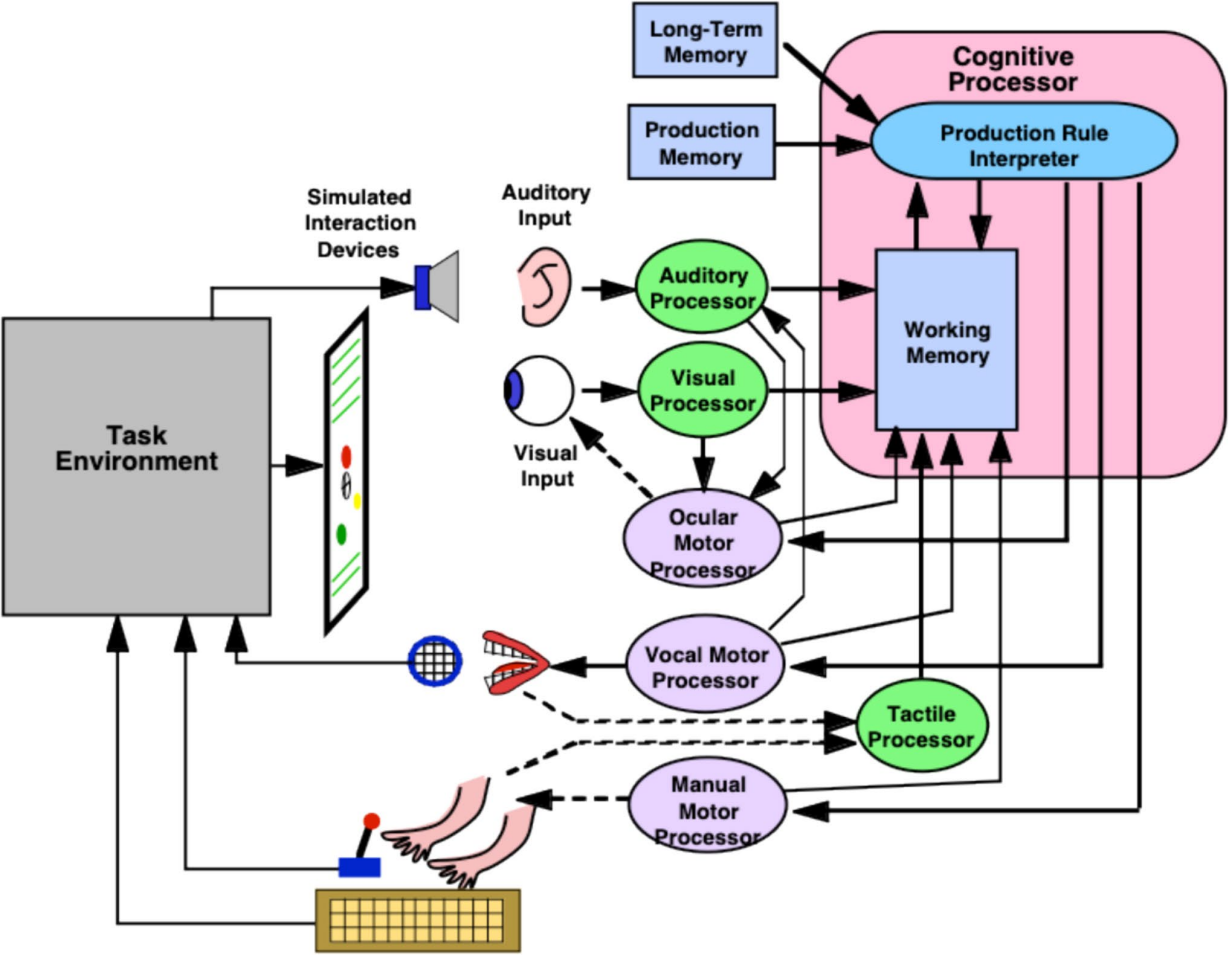
The EPIC architecture in simplified form. The simulated environment, or device, is on the left; the simulated human on the right. (Color figure online)

Figure 3 shows the overall structure of the EPIC architecture. In essence, EPIC provides a general software framework for simulating a human interacting with an environment to accomplish a task. The environment is represented as a *device* that provides simulated sensory input to the simulated human and responds to simulated motor actions from the simulated human. The simulated human consists of software components for memory systems, perceptual processors, and motor processors, surrounding a cognitive processor that executes a strategy, represented as a set of production rules, to perform the task. The device and these processors are all simulated to operate in parallel with each other.

To simulate human performance in a task, a modeler must specify the production-rule strategy for performing the task and may also set parameters for the perceptual and motor processors. When the simulation is run, the architecture generates the specific sequence of perceptual, cognitive, and motor activities required to perform the task. Monte-Carlo runs of the simulation produce actual behavior sequences for each trial, which like the human data, are aggregated statistically to produce predicted dependent variable values such as mean RTs and ERs in each experimental condition.

### The cognitive processor

As shown in Fig. 3, EPIC’s cognitive processor comprises two interconnected components, the production-system working memory (PSWM) and a production-system interpreter that executes the production rules for the task strategy. The contents of the PSWM consist of the output of the perceptual processors, the state of the motor processors, and the state of the strategy being executed. As the production rules are executed, movement commands can be sent to the motor processors, and the contents of PSWM can be modified.

#### **Production rules and cyclical operation**

A production rule consists of a condition and a list of actions. The condition describes information items that might be present in the PSWM. The actions can command motor processors and add or remove items from the PSWM.

The cognitive processor runs on a cyclic basis with a 50 ms cycle duration that is not synchronized with other events in the simulation. At the beginning of each cycle, after any pending modifications to PSWM are made, the conditions of all the rules are tested in parallel against the contents of PSWM, and those whose conditions match are “fired” (i.e., their actions are executed at the end of the cycle). The actions can modify the contents of PSWM, which may change which rules will match on the next cycle, or the actions can instruct motor processors to carry out hand, speech, or eye movements. The total number of production-rule cycles required to produce a response contributes to the time to complete the task.

#### Source of task strategies

A fundamental assumption of EPIC and similar production-rule cognitive architectures is that participants in experiments create a set of task-strategy production rules when first instructed about the task. They then presumably refine these rules as more experience is gained in performing the task. Feedback or incentives may play a role in how the task strategy is refined. However, EPIC does not attempt to represent any underlying learning mechanisms per se. Rather, the amount of practice is assumed to be extensive enough that participants have developed a stable strategy representable in terms of production rules. The modeler writes the production rules for an assumed stable strategy and then runs the model to produce the simulated behavior.

### Primacy of perceptual-motor constraints and task strategies

Meyer and Kieras (1997a, p. 14; Kieras, 2016) proposed that models of human performance should always incorporate the relevant known perceptual and motor constraints, with cognition providing only a mechanism for explicitly representing and executing a task-specific strategy with no a priori hypothetical processing capacity limitations. Thus, EPIC’s assumptions about cognition are essentially as parsimonious as possible. They ensure that known perceptual-motor constraints are taken into account rather than obscured by assumptions of central limitations.

Next, we describe the specific EPIC mechanisms for our models of simple visual search in this article. In particular, the following section presents early-vision and oculomotor mechanisms, and the basic task strategy used in these models.

## Specific EPIC mechanisms relevant to simple visual search

As previewed above, three types of EPIC mechanism are especially relevant for modeling simple visual search. They include visual mechanisms with early-vision limitations, oculomotor mechanisms that govern eye movements, and cognitive mechanisms that implement production-rule task strategies.

### Visual mechanisms

#### Visual objects and stores

The visual processor shown in Fig. 3 contains a set of stores and processors that construct and maintain representations of visual objects. In what follows, each component is described in the order that the information flows. *Visual objects* are essentially bundles of properties that share a spatial location and extent, both measured in degrees of visual angle. The spatial location of objects, and eye fixation location, are given in terms of coordinates whose origin is defined as “straight ahead.”

The *physical store* represents the current visual environment, such as what is on the display screen. Changes in the state of the physical visual environment are sent to the *eye processor*, which represents the retinal system and how the visual properties of objects in the physical store are differentially *available* depending on their physical properties, such as color and shape, and their *eccentricity*—the distance in degrees of visual angle from the center of the fovea (see review in Findlay & Gilchrist, 2003). The resulting “filtered” information is sent to the *sensory store*, where it persists for a fairly short time and comprises the input to the *perceptual processor*, which performs the processes of recognition and encoding. The output of the perceptual processor is deposited in the *perceptual store*, which is essentially visual working memory. This processing is done in parallel for all visual objects and their available properties, and for the visual properties in these models, is assumed to require EPIC’s default total of 50 ms. If the eyes move or the physical objects appear, disappear, change location, color, or size, the visual perceptual store will eventually be updated to reflect the current visual situation. The contents of the perceptual store are mirrored in the PSWM, which means that production rules can respond to this constantly updated representation of the current visual environment.

The perceptual store thus integrates over eye movements and maintains a cohesive representation of the current visual situation—corresponding to our subjective experience of a continuously present and integral visual surround. The appearance or disappearance of an object, or changes to its properties (such as its color or location), will be quickly updated in the perceptual store, but if the information is no longer supported by visual input due to an eye movement away from the object, the information persists for some time, on the order of seconds (see Henderson & Castelhano, 2005; Kieras, 2009b).

A fundamental property of EPIC’s visual system is that it has no built-in limit to the number of objects or their properties that can be held in the visual perceptual store. However, the position of the eyes, early-vision limitations, and elapsed time determine which objects and properties are present in the visual perceptual store, so the total information present is de facto limited.

#### Early-vision limitations in EPIC

Because the perfect early-vision assumption is abandoned in EPIC, the architecture must characterize early-vision limitations in detail adequate to support quantitative model predictions.

##### **Visual availability**

Availability, similar conceptually to acuity, refers to whether a particular visual property of an object, such as color or shape, can be perceived or detected as a function of *both* its *location* and *size* on the retina. Including visual extent along with eccentricity enabled EPIC models to fit visual search results in which the size of display objects varied in addition to their color and shape (Kieras, 2010). Extending the eccentricity results in Bouma (1970), Anstis (1974) provides some example measurements and comparisons showing that a single letter can be identified in the periphery if it is large enough. For example, the recognition threshold size of a single letter is about 0.2° at eccentricity of 5°, and about 1.3° at 30° eccentricity. Moreover, various visual properties are differentially available in peripheral vision; for example, color can be very available out to 40° or more (e.g., Gordon & Abramov, 1977).

A long-proposed neural mechanism for this relationship between eccentricity and size is *cortical magnification*: a constant amount of visual cortex (presumably supporting a certain number of receptive fields) is required for performing discrimination at a certain level. Anatomically, the density of cortical representation declines with distance from the fovea. Therefore, to maintain discriminability, the size of the stimulus must increase with eccentricity in order to involve the same amount of cortex. Such cortical magnification functions have been measured in psychophysical experiments; a typical result (e.g., Virsu & Rovamo, 1979) is that to maintain discriminability, the required size increases roughly linearly up to a moderate eccentricity and then quite sharply in the further periphery, with a cubic function providing a good fit.

Unfortunately, the available psychophysical and visual search literature does not use a standard set of visual properties. For example, the orientation property is popular in visual search experiments, but the exact manipulations of orientation are tremendously varied; the stimuli range from very short line segments slightly tilted left or right, to very large horizontal and vertical bars as in the Wolfe et al. (2010) experiment modeled here. Thus, we lack a set of empirical parametric functions that describe the availability of common object properties.

##### **Representing availability**

EPIC’s visual processor uses *availability functions* of a certain form based on the psychophysical literature, and given the deficiency in the empirical literature, the parameters of the functions are estimated to fit the data being modeled. However, extant psychophysical results do set some constraints—for example, the color of an object of a certain size and eccentricity is expected to be more available than its orientation, and much more available than its detailed shape. In addition, rather than the cubic function in the cortical magnification results, a simpler function is suggested by the results in Anstis (1974) that show a linear relationship between detection threshold for object size and eccentricity up to 30° (cf. Bouma, 1970). The Wolfe et al. (2010) displays have eccentricities in this range, so the models in this article use a simple Gaussian detection function that gives the probability that a specific property will be available (i.e., detected) for an object with size *s* at eccentricity *e*:

$$P(detection) = P(s>N(\mu , \sigma )), \mu =\uptheta e, \sigma =0.5$$

The value *μ*, the mean of the Gaussian function, can be interpreted as the 50% detection threshold in terms of the object size. It is the product of eccentricity and the parameter θ which is the *availability threshold coefficient*. Like thresholds in general, small values of θ correspond to the property being *more* available (lower threshold, more detectable at a given eccentricity and size), and large values of θ mean that the property is *less* available (higher threshold, less detectable at a given eccentricity and size). The standard deviation *σ* determines the “steepness” of the detection function and was held constant at 0.5. Thus, only the θ parameter was adjusted to fit the observed data for the different properties. For simplicity, the specific values for each property are assumed to have the same availability (e.g., a Color property value of Red is assumed to have the same θ as a Green value). The size of the objects used in these displays is defined as the average in degrees of visual angle of the vertical height and horizontal width of the bounding box of the object.

**Fig. 4:**
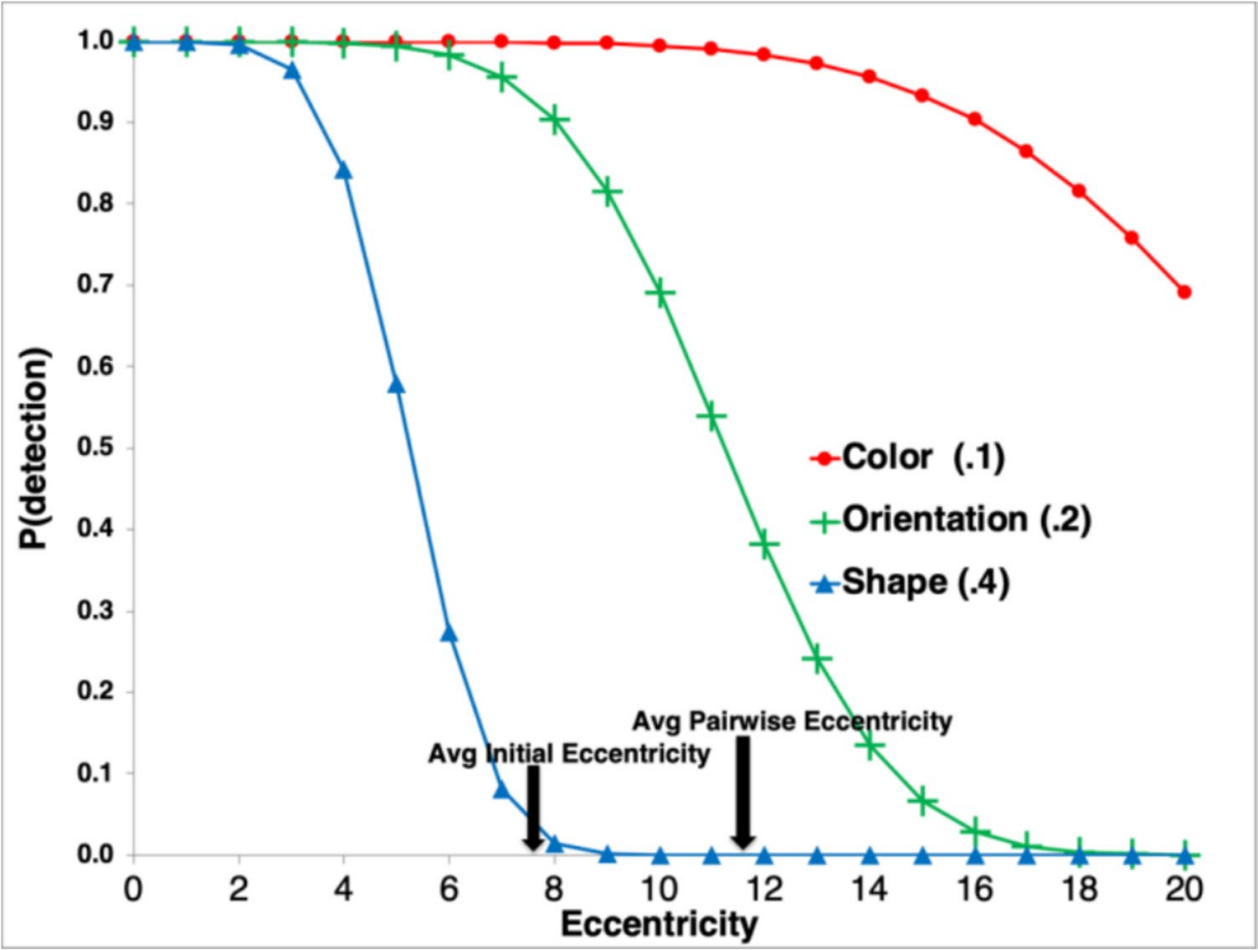
Example availability functions and threshold coefficients for the Color, Orientation, and Shape properties used in the simple visual search task. (Color figure online)

Figure 4 shows the availability functions for some representative values of θ used in models for the Wolfe et al. (2010) data presented later in this article—namely, θ_C_ = 0.1 for Color, θ_O_ = 0.2 for Orientation, and θ_S_ = 0.4 for Shape. In addition, Fig. 4 shows two useful values for the average eccentricities of display objects in the modeled tasks. These values were computed from a large sample of displays generated by our simulation of the tasks (described below). The *average initial eccentricity* of the objects is based on the eyes looking directly at the specified initial fixation point of the display when the objects appear. The *average pairwise eccentricity* is the average distance, for all possible fixated objects, between the fixated object and all the other objects.

As shown in Fig. 4, the Color property with its small θ threshold coefficient is highly available; its detection probability is high throughout the eccentricity range. Orientation, whose θ is larger than for Color, is substantially less available; at the average pairwise eccentricity, the probability of correct detection is only about 0.4. Finally, Shape, with a large θ, is not very available at all; even at the average initial eccentricity, the probability of detection is quite low. Thus, fixations within a few degrees of an object will be required to reliably detect its Shape.

##### No bottleneck in availability for multiple objects

With EPIC’s visual system, the properties of more than one object can be available at the same time in the perceptual system for a given eye location, and all this information is available simultaneously. No “attentional” or “capacity” limit is assumed to prevail at this level of the visual system. If the availability is high enough (a low θ threshold coefficient), then the properties of many objects, even at large eccentricities, may be available.

##### Relation to functional visual field

This feature of EPIC’s visual system is similar to the long-standing concept of the *functional visual field* (FVF; Hulleman & Olivers, 2017; also see Wu & Wolfe, 2022). They both allow more than one object to be perceived in a single fixation. However, the FVF concept assumes there might be other factors that affect the processing of objects covered by a fixation, including possible attentional limitations.

In contrast, the EPIC availability functions determine only whether the visual properties of an object are available for further processing given the object’s size and eccentricity, independent of any other factors. In addition, availability is defined as a continuous probability function over the entire visual field, and each property has its own availability function reflecting how it is detected by the visual system.

##### **Availability and eye movements**

The availability of each property is independently sampled for all objects when the display first appears and resampled whenever the eyes are moved. As the eyes move around, the available properties of a particular object can fluctuate, and will not necessarily be available from one fixation to the next. However, the properties, once acquired, will remain for some time in the visual perceptual store, where production rules can match objects that have the properties relevant to the task.

##### Crowding

Crowding refers to the phenomenon in which the perception of an object is impaired if it is surrounded by closely spaced objects, but the same object is perceived accurately if the spacing is larger or the eccentricity is smaller.

Crowding in the recognition of characters in reading was described by Woodworth (1938) and then later by Bouma (1970), as well as Anstis (1974). Pelli and Tillman (2008a, b) provide many demonstrations of the effect for a variety of stimuli. Levi (2008), Pelli (2008), and Rosenholtz (2016) argue that crowding is essential to understanding extra-foveal vision because it appears to be more responsible for the limitations on peripheral vision than mere loss of resolution.

Crowding effects appear if the center-to-center spacing between the objects is less than a *critical spacing*, which for a variety of visual properties turns out to be approximately half the eccentricity of the object in question, a relationship first reported by Bouma (1970). Thus, crowding would impair the accurate perception of a set of closely spaced objects if the eccentricity is large, but by moving the point of fixation close enough, the critical spacing becomes smaller, allowing the adjacent objects to be perceived correctly.

##### **Crowding in simple visual search**

Simple visual-search experiments *almost always confound the number of objects in the display with object spacing*. Typical experiments have varied set size while placing the objects at random within a constant display area, thereby producing higher average object density for larger set sizes. The few studies attempting to separate crowding and set size effects suggest that much of the reported set size effects in simple visual search could in fact be due to crowding rather than set size (e.g., Motter & Simoni, 2007, 2008; Wertheim et al., 2006). Regardless of this possible confounding, Wolfe et al. (2010) simply asserted that the stimuli “can be easily identified outside of the fovea” (p. 1305) without providing any measurements showing that this was true for their higher-density displays.

To assess whether crowding might be confounded with set size in the Wolfe et al. (2010) displays, we calculated how much crowding occurred for a large sample of displays randomly generated by our simulation of the tasks. If the eyes are assumed to be at the initial fixation point, the average number of crowding objects (within the critical spacing) for each display object increases from 0.1 at set size 3 up to 1.1 for set size 18. If the eyes are assumed to fixate each object, the average number of crowders for each other object increases from 0.15 at set size 3 to 2.5 at set size 18.

Thus, although set size was the intended manipulation, the experimental paradigm of Wolfe et al. (2010) apparently also manipulated a confounded variable, the amount of crowding.

##### **A simple crowding mechanism**

Based on the literature (e.g., Keshvari & Rosenholtz, 2016; Levi, 2008; Pelli et al., 2004; Pelli & Tillman, 2008a, 2208b; Põder & Wagemans, 2007; Rosenholtz, 2016; Rosenholtz et al., 2019; Strasburger, 2020; Yashar et al., 2019), it is reasonable to assume that the perceptual features of the crowded and flanking objects may get attached to the wrong objects—essentially *scrambled* between the objects that crowd each other. Hence, for the Wolfe et al. (2010) displays, which consist of simple discrete objects and properties, we implemented a very simple form of feature scrambling.

During this process, the properties of the visual objects are determined in two steps that take place whenever the display objects initially appear or the eyes are moved. First, the visual system applies the availability functions to determine which properties are currently available for each object given the current eye position; unavailable properties are assigned *blank* values. Second, the crowding mechanism randomly scrambles the values of each property, including blank values, between objects in each *crowding group*, which are the objects within the critical spacing of each other. To parameterize the amplitude of the crowding effect, the scrambling of a property is performed with a certain *crowding probability* ϕ when each object is processed. The value of ϕ can differ depending on the property but is assumed to be the same for all values of that property. The crowding probability ϕ applies to each object. For example, a crowding group of four objects might have scrambling applied a total of four times, once for each of the objects.

As explained below, the models can treat Shape as a unitary property like Color and Orientation. Thus, the same scrambling mechanism for crowding applies to Shape as well as Color and Orientation. Representative estimated values for the models in this article are ϕ_C_ = 0.025 for Color, ϕ_O_ = 0.025 for Orientation, and ϕ_S_ = 0.1 for Shape.

#### Early-vision limitations produce illusory targets, distractors, and blanks

The availability mechanism and then the crowding mechanism are applied when objects first appear in the visual display. These mechanisms are also applied again after each eye movement. Consequently, as the eyes are moved around the display, the perceived objects tend to acquire properties in the perceptual store, either from properties becoming available due to nearby fixations, or because properties are scrambled between nearby objects.

During this process, a property value for an object may be replaced by some other object’s property value. Consequently, three cases can result: (1) The representation of a distractor object might get a target property and thus become an *illusory target*. (2) The representation of the target object might get a nontarget value, becoming an *illusory distractor*. (3) The representation of the target object might get a blank property and thus become an *illusory blank* even though its actual property value would be available in the absence of crowding. The presence of these illusory objects can affect the outcome of a visual search strategy.

### Motor mechanisms

#### Eye-movement time and accuracy

As indicated in Figure 3, production-rule actions can command the *ocular motor processor* to make a saccadic eye movement to the location of a designated object represented in the perceptual store. Various studies have shown that saccades tend to fall short of the actual fixation target, and the standard deviation of the saccade length tends to be proportional to the length (Abrams et al., 1989; Harris, 1995). Thus, the oculomotor processor samples the length for a saccade to an object at eccentricity *e* from a Gaussian distribution:

$$saccade\, length = N(\mu , \sigma ), \mu =g\bullet e, \sigma = s\bullet \mu$$

Typical empirical values for *g* (*gain*) range from 0.85 to 0.95, and *s* (*spread*) is typically around 10%. Harris (1995) obtained estimates of *g* = 0.95, *s* = 10%, which are consistent with observed values of these parameters. These values are used in EPIC as the default values for oculomotor noise along the line of flight of the saccade.

Angular error might also be present; a saccade might not only fall short, but it might also miss to one side. Unfortunately, there are very few studies on angular error; a simplified model inspired by van Opstal and van Gisbergen (1989) samples the saccade polar angle from a Gaussian distribution whose mean is the actual angle and whose standard deviation is a constant, currently defaulting to simply 1°.

The time duration of a saccade was determined using the classic linear function described by Carpenter (1988):

$$saccade duration(ms) = 21 + 2.2\bullet saccade length(degrees)$$

##### Manual motor time and accuracy

The time to make a key-press response is represented by an architectural mechanism first proposed in EPIC for use in models of the psychological refractory period (Meyer & Kieras, 1997a, 1997b). In these high-speed tasks, the participant has a finger poised over each response key so only a rapid finger flexion is required. The manual motor processor uses a motor programming concept whereby producing a movement of this type requires selecting motor features that specify the hand and finger, then initiating the actual movement, which takes a certain amount of time to close the key switch (see Kieras, 2009a, for additional discussion). Each feature was estimated as requiring 50 ms to select; the initiation time was also estimated at 50 ms, and the movement time at 25 ms. The motor features from a previous movement can be reused for an immediately subsequent movement, enabling it to be produced more quickly.

Wolfe et al. (2010) do not provide specifics about the response keys they used. We assumed that the responses differed in only one feature. Since present and absent responses were approximately equally probable, it is reasonable to suppose that the motor time on average for present and absent responses would be the same in terms of the average number of features reused. Thus, the time contributed by the manual motor processor was set at 125 ms for both present and absent responses in all task conditions and set sizes.

The models presented here account for error rate (ER) as well as RT. One component of ER is a low and constant rate of *action slips*, or “oops” errors, a basic error mechanism that is often postulated in human performance research (e.g., Norman, 1981). Namely, the participant has the correct intention for a movement, but at random, an incorrect motor action will be triggered, often one that is frequently made. This most basic source of errors can be represented very simply by assuming that the manual motor feature specifying the present/absent manual response might “flip” to a different and frequent value. Thus, when the strategy calls for a present or absent response, the *opposite* response is made with a slip error probability *SlipER*.

### Cognitive task-strategy mechanism

As mentioned previously, in the EPIC architecture, the only function of the cognitive processor is to execute a task strategy for supporting task-specific programmable behavior. The strategy is represented in terms of production rules as summarized above. For visual search tasks, the cognitive processor determines which rules have conditions that match the contents of the production-system working memory and then executes the actions of those rules. These actions have effects such as noting which objects are possible targets, moving the eyes to an object of interest, or making a key-press response.

This component of the architecture is thus a mechanism like those already presented. However, rather than setting numerical parameters to modify its behavior as in the perceptual mechanisms, the modeler devises production rules for specific strategies and loads them into the simulated cognitive processor to govern how the task is performed. In general, the choice of strategy has a large effect on whether an EPIC model can fit the data, and a satisfactory fit can only be obtained by a good choice of both perceptual-motor parameter values and strategy. Thus, the choice of strategy is conceptually similar to a parameter setting, but it is a qualitative and symbolic parameter, rather than a conventional numeric parameter.

More than one strategy was needed to account for how participants performed the various visual search tasks studied by Wolfe et al. (2010). The next section describes a basic strategy used as a starting point for our models. Subsequent sections describe *strategy variations* required to fit models for data from some of the different tasks.

It should be noted that production rules are essentially a programming language with detailed syntax and semantics specific to the production-rule interpreter implemented in a particular cognitive architecture. However, the essentials of the present task strategies can be captured in pseudocode. It is therefore unnecessary to provide technical details of the production-rule syntax (see Kieras, 2016) or the “control” and “housekeeping” production rules for performing a series of trials in succession.

#### The basic search strategy

**Fig. 5:**
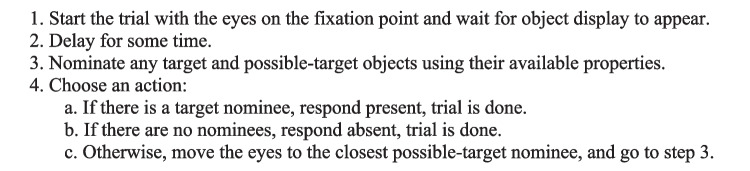
Pseudocode for the Basic Search strategy. Each application of Step 3 and Step 4 requires one 50-ms cycle. Steps 4a and 4b also require manual movement time. Step 4c also requires eye-movement time

The *Basic Search* strategy, shown as pseudocode in Figure 5, is a general strategy for performing simple visual search tasks. It involves four main steps. At Step 1, the eyes are placed on the initial fixation point and the model waits for the display objects to appear. Step 2 represents the start-up delay for the strategy, *VDelay,* held constant at 100 ms in all the models. Then during Steps 3 and 4, the strategy production rules alternate between two phases: In the Step 3 *nomination phase*, *nomination rules* fire to nominate objects in the visual field that are either the actual *target*, having all the required target property values, or *possible targets* because a relevant target property is blank (unknown). In the Step 4 *choice phase*, specific rules fire to take one of three actions: (a) If an actual target object has been nominated, a target-present response is immediately made via a command to the manual motor processor. (b) If there are no nominations at all, meaning that all objects appear to be distractors, then a target-absent response is immediately made. (c) Otherwise, there are only possible-target nominations, so an oculomotor-processor command is issued to move the eyes to the *closest* nominated object. Once the eye movement to this candidate is complete, the nomination phase starts again at Step 3.

The response time produced by this strategy increases with (i) the number of occurrences of Steps 3 and 4, and (ii) the total number of eye movements. The rules for Steps 3 and 4 in Fig. 5 each require a single 50 ms production-rule cycle.

The Basic Search strategy constitutes an *optimal strategy* that is essentially the “fastest reasonable” way to perform the task. It is “fastest” because it exploits extra-foveal vision: fixating each object may not be necessary. Thus, a target-present response is produced as soon as a target is detected, even if it has not been fixated. A target-absent response is produced as soon as all objects appear to be distractors, regardless of whether they have all been fixated. This strategy is “reasonable” because the response will be as accurate as possible given perceptual limitations and possible slips in the response motor action. The presence of illusory targets, distractors, and blanks will affect the accuracy and possibly the speed of the response, as discussed below. The RT depends primarily on how many eye movements are made during this process.

Many other visual-search models make a target-absent response after some time has elapsed without finding a target (see review in Hulleman & Olivers, 2017). However, the Basic Search strategy states simply that a target-absent response is made as soon as there are no target or possible-target nominations (i.e., *everything looks like a distractor*); no “time-out” branch in the strategy is required.

**Fig. 6:**
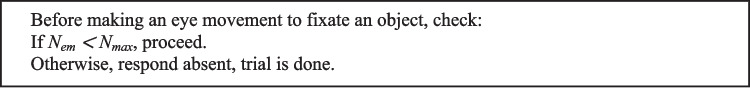
Pseudocode for the Limited-fixations strategy option

Yet there is a useful variation on the Basic Search strategy that enables a kind of “time out” effect. The *limited-fixations* strategy option, shown in Fig. 6, provides a way to speed up the search at the expense of accuracy. If the target has not been found, and *N*_*em*_*,* the number of eye movements made thus far in the trial equals or exceeds a limit, *N*_*max*_, an absent response is immediately made. In the Basic Search strategy (Fig. 5), this option would be placed at the beginning of Step 4c. Because Basic Search moves the eyes at a roughly constant pace, this option is a simple form of the popular “time out” stopping mechanism mentioned above. It can be placed in any variant of Basic Search before each step that initiates an eye movement.

#### Visual properties and search strategies

The Basic Search strategy nominates targets and possible targets based on the available properties of visual objects, which are governed by the availability functions and crowding mechanism. This implies some assumptions about the relevant visual properties for the Wolfe et al. (2010) tasks.

##### **Color and orientation**

It seems obvious that for the Color and Conjunction tasks, the relevant properties are the traditional Color and Orientation features, that if available, have one of two values. It also seems reasonable to assume that these properties are *unitary*, in that the values are available or unavailable in an all-or-none way.

Under these conditions, the nomination rules are very simple for the Color task because only a single object property is involved: An object is nominated as the target if it has a Red Color, or as a possible target if it has an unknown (blank) Color. If all objects are perceived with a Green Color, then no objects can be a target, so there are no nominations, leading to an immediate absent response.

In contrast, for the Conjunction task there are four possible nominations: a target nomination for a Red Vertical bar, and three different types of possible-target nominations—Red Color and blank (unknown) Orientation; blank Color and Vertical Orientation; blank Color and blank Orientation. If a target has been nominated, a present response is made; if one or more possible-target nominations are present, the strategy will choose one to fixate in the descending priority order as just listed, which gives priority to Color if it is available (cf. Kaptein et al., 1995). Finally, if there are no nominations because all objects appear to be either Horizontal Red or Vertical Green, an immediate absent response is made

##### **Shape as a unitary property**

That Color and Orientation are unitary properties seems reasonable, but the strategies also treat Shape as a unitary property, such that the possible values are simply *2*, *5,* or *blank*. This claim seems counterintuitive, given that these shapes appear to have a detailed substructure of line segments so that the actual shape could be partially available rather than all-or-none. Defining these possibilities is difficult. However, the proposed search strategies enable a great simplification because they allow the apparently complex Shape property to be meaningfully treated as unitary. This follows from the fact that “partial” encodings of the Shape match neither a target nor a distractor. Consequently, they can simply be treated as *possible targets*, just as if they had a blank Shape property.

The Shape task can thus be modeled as a single-feature task with nomination and choice rules that are as simple as those for the Color task: If a *2* is visible, a target is nominated and a target-present response is made; if a *blank* is visible, it is a possible-target nomination. If all objects appear to have a *5* shape, then no nominations are made, and the response is “target absent.” The availability of Shape can thus be represented with a detection function whose threshold θ_S_ is higher than that presumably involved with detecting an individual hypothetical subfeature. Crowding will scramble these unitary Shape property values according to the same algorithm as for Color and Orientation.

#### How the visual search strategy makes errors

As will be pointed out in our discussion of the Wolfe et al. (2010) results, there are clear systematic effects in the ER data as well as the RT data, and both should be accounted for as equal-status measures of visual search processes. The Basic Search strategy will produce correct responses unless the perceptual information, or the motor response, is inaccurate. Thus, errors result only from the following four sources in the models to be presented:

##### **Illusory distractors from crowding**

Miss errors result when the strategy rule that detects the absence of nominations fires although the target is in fact present on the display. This would happen if crowding scrambling turned the target into an illusory distractor. Thus, misses would increase with set size due to more crowding, and possibly with less available properties.

##### **Illusory targets from crowding**

False-alarm errors could occur if the strategy rules detect the presence of the target when there are only distractors on the display. For example, this might happen if crowding scrambled distractor features into an illusory target, which seems especially likely in the Conjunction task because many instances of each property value are on the display.

##### **Premature termination of search**

The limited-fixations strategy option (Fig. 6) terminates the search with an absent response after a certain number of eye movements. Thus, a miss error will result if the strategy terminates a positive trial with an absent response before the target has become available.

##### **Action slips**

The presented strategies will not “deliberately” respond present on a negative trial, so false-alarm errors must be due to some source other than the visual and strategy mechanisms. These errors are attributed to the *action slips* or “oops” errors described in the previous section on manual motor mechanisms. In these models, if the strategy calls for a present or absent response, the *opposite* response is made with probability *SlipER*. This will produce both false alarms and misses, but with a constant probability across search tasks, trial polarity, and set size. Note that these slip errors do not affect the correct trial RT distributions.

Thus, the logic of the Basic Search strategy together with a combination of crowding effects, limited fixations, and action slips could account for some basic features of the ER results shown in the next section. Especially consistent with this account are the increase of misses with display set size and apparent task difficulty. The fact that false-alarm rates are low and very stable in the data suggests that illusory targets either occur rarely or the task strategy prevents them from governing responses, leaving action slips as the only source of false alarms.

## Creating the EPIC models for simple visual search

Now that the relevant EPIC mechanisms have been introduced, this section presents the experimental results of Wolfe et al. (2010) and describes how EPIC models were constructed and fit to their RT and ER data. We chose these data to be modeled because of their very high quality, which is due to the relatively well-specified stimuli and very large number of trials from very well-practiced participants.

### The experimental procedure and empirical data

Modeling the Wolfe et al. (2010) data in Fig. 2 required simulating not only the mental processes of the human participants, but also the Wolfe et al. (2010) experimental materials and procedure. Doing so involved making some plausible assumptions about details that Wolfe et al. (2010) did not report. In addition, we reanalyzed their data, including both RTs and ERs. Even though we did not conduct the experiment ourselves, the following subsections use the familiar Method and Results organization to simplify the presentation of both our assumptions about procedural details and our reanalysis of the data.

#### Method

##### **Tasks**

There were three different present/absent simple search tasks. Figure 1 shows a sample target-present display produced by the simulation for each task condition.

The three tasks were as follows. *Shape task*: The objects are “digital *5*” and “digital *2*” shapes formed from vertical and horizontal line segments similar to these digits on a traditional seven-segment digital display. The distractors are always *5*s; if present, the single target is always *2*. *Color task:* The objects are vertical bars. The distractors are always green. If present, the single target is always red. *Conjunction task:* The objects are bars that are vertical or horizontal, and red or green. Half of the distractors are green verticals and half are red horizontals. If present, the single target is always a red vertical bar.

##### Construction of stimulus displays

Wolfe et al. (2010) do not describe exactly how the displays for individual trials were created. Their downloadable dataset specifies for each trial the polarity and set size, but not the actual specific display used in that trial. Thus, for purposes of modeling, our simulated task environment generated the display for each simulated trial with the process described in what follows. The example displays in Fig. 1 above were generated by the task simulation.

According to Wolfe et al., the search display was an area whose visual angle extent was 22.5° × 22.5°, containing 25 invisible cells of 5° × 5°. In the simulation, the location of an object was defined to be the center point of the bar or digit bounding box. Wolfe et al. state that each object appeared in a random location within one of the cells, but they did not state whether or how touching or overlapping objects were prevented. Assuming that such displays were not allowed, the random location within a cell was constrained in the simulation to keep the horizontal or vertical edge of the bounding box for an object at least 0.25° away from the cell boundary, ensuring a minimum separation of 0.5° between edges of adjacent objects.

Set sizes were 3, 6, 12, and 18. In the simulation, a display was generated for each trial as follows: the set-size number of distractors were first placed in randomly chosen display cells. With probability of 0.5, the trial polarity was then determined; if the trial was positive (target present), a randomly chosen distractor was replaced with a target object.

According to Wolfe et al., in the Shape task, the objects were 1.5° × 2.7° character-like shapes. In the simulation, the visual size of an object was defined as the average of the horizontal and vertical bounding box dimensions, giving 2.1° for the defined Shape object size. The target was a *2* and the distractors were *5*s. In the Color task, the objects were 1° × 3.5° vertical bars (size defined as 2.25°); the target bar was red, distractor bars were green. In the Conjunction task, the objects were also 1° × 3.5° bars, red or green, oriented either horizontally or vertically. The target was a red vertical bar, and distractors were red horizontal and green vertical bars. In the simulation, half of the distractors were chosen to be of each type. Set size 3 was special-cased so that at least one distractor of each type was present on each trial, so that averaged over trials, each type of distractor would appear equally often.

##### Design

There were 10 participants in the Conjunction task condition and nine in the other two conditions. One individual participated in both the Conjunction and Shape tasks. However, the dataset does not identify this participant, so in our statistical analysis, the task condition was treated as a purely between-participant manipulation.

##### **Procedure**

Each trial began with a centered fixation cross. Participants were instructed to “keep their eyes focused on this cross” (Wolfe et al., 2010, p. 1306). However, because eye movements were not monitored, participants could have moved their eyes, and as pointed out above, a better starting assumption is that they did so. The search display was presented and remained visible until the participant pressed a key for target present or target absent.

Participants were instructed to respond “as quickly and accurately as possible” (Wolfe et al., 2010, p. 1306), and correct/incorrect feedback was presented for 500 ms after each trial. However, no explicit incentive such as a payoff function (Edwards, 1961; Sternberg, 2016, Appendix B) was provided to make the task instructions more specific or implementable by the participants.

Nevertheless, unlike in many visual search experiments, the participants were very well practiced. They completed 13 blocks of trials, with 30 practice trials at the beginning of each block. There was a total of 4000 experimental trials per participant, with set size and polarity randomly determined on each trial. This yielded about 500 trials per participant for each combination of set size and positive/negative trial polarity.

#### Results

The data collected by Wolfe et al. (2010) are publicly available for download at Visual Attention Lab  n.d.). The downloaded data consisted of the RT and correct/incorrect response status from each trial for each participant at each set size and trial polarity. RT outliers were removed from the data according to the description in Wolfe et al. (2010). Following common practice in RT experiments, the data were reduced as follows.

For each participant in each task condition, we calculated the mean RT of correct responses and the proportion of errors (error rate, ER) on positive and negative trials at each set size, giving a total of 8 mean RT data points and 8 mean ER data points for each participant. These participant means were then averaged to produce the data points plotted in Fig. 2, above. Throughout this article, positive (target present) trials are shown as red points and lines, with negative (target absent) trials in black; the Shape task is plotted with squares, Conjunction with triangles, and Color with circles. The 95% confidence intervals around each data point in Fig. 2 were calculated by determining the standard error of that mean using the nine or 10 individual participant means contributing to that point, thus reflecting between-participant variability, but not within-participant variability.

Wolfe et al. did not report any overall statistical tests for these results. Therefore, we performed unequal-*n* analyses of variance (ANOVAs) on the reduced data using the R *ez* package (Lawrence, 2016; R Core Team, 2017). For RT, the main effects of task condition, trial polarity, set size, and all two- and three-way interactions were significant (*p* <.05). For ER, whose overall average was 2.4%, the task condition main effect was not significant (*p* >.1) but the trial polarity and set size main effects, and all two- and three-way interactions were significant (*p* <.05).

Table 1 presents some summary statistics from these results. Given the great importance in the literature attached to the linearity and slope of the RT functions, this table provides the intercept, slope, and *r*^*2*^ of a linear fit to the mean RT data (ms) in each condition and trial polarity, along with the ratio of the negative-trial RT slope to the positive-trial RT slope. Since the Color task slopes are essentially zero, the slope ratio is not meaningful in this condition. Also shown is the mean ER and the maximum ER in each condition.

**Table 1:** Summary statistics for Wolfe et al. (2010) data

Task	Negative trials				Positive trials					
	Intercept	Slope	*r*^*2*^	ER	Intercept	Slope	*r*^*2*^	ER	MaxER	Slope ratio
Color	436	−1	0.68	0.014	395	1	0.90	0.025	0.031	−0.69
Conjunction	480	26	1.00	0.014	483	9	1.00	0.032	0.049	2.84
Shape	589	95	0.99	0.014	574	43	0.99	0.045	0.093	2.21

### Discussion

#### Reaction times

The reaction-time data from Wolfe et al. (2010), shown in Fig. 2 and summarized in Table 1, follow the classic pattern obtained in most simple visual-search experiments. The RT functions are essentially flat in the Color task (positive trial slope is about 1 ms/item). In the Conjunction and Shape tasks, positive and negative trial RTs have a substantial slope, with the negative-trial slope being roughly twice or more than the positive-trial slope, which is the classic indicator of a serial self-terminating search.

#### Error rates

As described above, our analysis of the ER data showed that all main effects and two- and three-way interactions are strongly significant despite large individual differences. There are very few errors on negative trials (false alarms), and their rate depends very little on the task condition and set size (actually declining slightly with set size). The average false-alarm rate across all task conditions and set sizes is only 1.4%. In sharp contrast, the errors on positive trials (misses) are overall more frequent than the false alarms, and strongly depend on the task, being lowest in the Color task and highest in Shape. The miss rate in the Conjunction and Shape tasks strongly increases with set size but is essentially constant in the Color task. Thus, these ER data are highly systematic.

Error-rate effects in the literature on simple visual search typically have been ignored when *overall* ERs were low and positively correlated with overall RTs, meaning that an overall speed–accuracy trade-off was not present. Under such conditions, both Wolfe et al. and other past investigators have focused only on the RTs from correct trials (cf. Pachella, 1974).

Yet despite this conventional justification for ignoring ER effects, in the data from Wolfe et al. (2010), they are pronounced, systematic, and statistically reliable, and therefore deserve to be explained along with the RT effects. Furthermore, rather than simply postulating that ER effects depend on error rate parameters that increase with task difficulty, it would be better to explain them in terms of the same visual and strategy mechanisms used to explain the RT effects. Thus, in what follows, ER is considered as a first-class dependent variable along with RT for constructing and evaluating models of simple visual search.

### Constructing the EPIC models

#### Cognitive simulation models based on explanatory sequences

The present models were constructed and evaluated in a highly systematic way that itself constitutes a useful contribution. Work on cognitive simulation models in psychological research has usually presented only a final good-fitting model. This has motivated a classic criticism that the good fit resulted from the models simply having “so many degrees of freedom.” Here we instead show that the present final good fits stem systematically from a particular minimum necessary combination of explicit architectural mechanisms, parameter values, and task strategy.

To meet this goal requires rigorously considering models that *fail* to fit the data because they lack the necessary mechanisms, parameter values, or strategy.^2^ An elegant and logical way of doing so proceeds through *explanatory sequences* of models that show the effects of successively adding mechanisms, parameter adjustments, and strategy variations—*one after another—*to demonstrate how each contributes specifically to an increasingly better fit. Presenting an explanatory sequence culminating in a final best model is lengthy. However, the sequence clearly establishes that the final model is neither arbitrary nor haphazard; rather, it is really the best explanation of the data in terms of the underlying architectural assumptions.

##### **Constructing the explanatory sequences**

Our approach starts with an initial model that uses the fewest and simplest mechanisms to perform the task: these include the perceptual-motor mechanisms with parameter values based on previous literature and a minimal adequate task strategy. We add mechanisms and adjustments in a *priority order* motivated by our principle of perceptual-motor and strategy primacy mentioned above. First, we try to fit the initial model of an explanatory sequence by varying its perceptual-motor parameters. Next, if necessary, we modify the task strategy. If these combined efforts do not yield an excellent fit to the data, then we add or modify the perceptual-motor mechanisms and try again. Only after all these efforts fail would we have reason to add a cognitive mechanism beyond simple strategy execution, such as a “central” cognitive bottleneck or covert-attention shifting. As a result, EPIC models constructed through the explanatory-sequence approach do well at accounting for diverse phenomena without postulating unnecessary hypothetical cognitive mechanisms.

#### Generating and evaluating model predictions

##### **Model implementation**

Our first versions of the models for simple visual search were implemented using the current EPIC architecture software system. However, when the role of crowding became evident, it was necessary to explore alternative implementations for crowding, which led to the simple mechanism described above. To facilitate this process, a C++ “clone” of full EPIC model was constructed and verified, and then the crowding implementation was developed in the C++ model. The C++ versions of the EPIC models were then used for obtaining the model fits reported in this article because their great run speed made it convenient to generate *1 million* simulated trials for each combination of task condition, set size and trial polarity All the reported simulation results are based on this sample size. For the source code and data files for this model, see Kieras (2025).

##### **Metrics tor evaluating goodness-of-fit**

A model’s goodness-of-fit is often described in terms of a single metric, usually *r*^*2*^. However, for present purposes, the EPIC models’ goodness-of-fit will be described not only by *r*^*2*^, but also by two additional metrics reflecting the average absolute difference between predicted and observed values: namely the average absolute relative error (*aare*) and the average absolute error (*aae*). Together, the three metrics (*r*^*2*^, *aare* and *aae*) let us more completely characterize the relationship between predicted and observed dependent variables such as RTs and ERs; each metric conveys both useful information and corrects for misleading information sometimes conveyed by the other metrics.

## EPIC models for the aggregate data

All the relevant theoretical and methodological concepts for constructing and evaluating EPIC models of simple visual search have now been introduced.

The remaining sections of our article describe how these models account successfully for the data from Wolfe et al. (2010). In particular, EPIC models for their aggregate data are presented next. To demonstrate how the explanatory-sequence approach ensures that the model mechanisms are both necessary and sufficient, we present in detail the complete sequence of models for the Shape task. After that, for brevity, we only summarize the explanatory sequences for the Color and Conjunction tasks. More detail can be found in Kieras (2020).

### Explanatory sequence of models for the Shape task

We consider the Shape task first because, of the three tasks investigated by Wolfe et al. (2010), it is the most difficult. The Shape task requires extended nontrivial search over multiple seconds of time, and there is a large increase in the mean RTs as the number of displayed objects increases.

#### Step 1. Use the basic search strategy and vary availability to fit RT slopes

This explanatory sequence starts by our attempting to fit the RT slopes using the Basic Search strategy combined with mechanisms of visual availability plus eye and hand movements. Recall that a covert-attention shifting theory was originally proposed for explaining why RT slopes were too small to stem from eye movements that look at each object. However, our Step 1 shows that these slopes can be obtained if the properties of multiple objects are available during each eye fixation. Thus, the visual availability parameter will determine how many eye movements occur before the strategy chooses a response, which then determines the predicted RT.

**Fig. 7:**
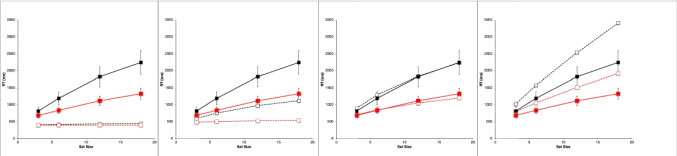
Shape task: Decreased availability produces larger slopes using Basic Search. Observed RTs and predicted values using Basic Search and varying availability. Left to right: θ_S_ = {0.1, 0.2, 0.4, 0.6}. RT scale 0–3,500 ms. Red: positive trial RT; Black: negative trial RT. Observed: Solid points and lines; Predicted: Open points and dotted lines. (Color figure online)

Figure 7 depicts the predicted and observed mean RTs as the availability threshold coefficient θ_S_ ranges from 0.1 in the leftmost panel (where the shapes of almost all displayed objects would be available without any eye movements) to 0.6 in the rightmost panel (where very few of the objects’ shapes would be available from any given fixation point; cf. Fig. 4). Predicted RT values appear on the graphs as open points and dotted lines, while observed values appear as solid points and lines (a convention used throughout the remainder of this article).

These fits from left to right bracket the observed RTs. The predicted and observed ERs are not shown here because for this model, the predicted ER is zero throughout, which will be addressed in the next explanatory step.

As Fig. 7 shows, when availability decreases, the predicted slopes increase due to an increase in the number of eye movements generated by the model. In the leftmost panel with highest availability (θ_S_ = 0.1), almost no eye movements are made, resulting in fast and almost flat predicted RTs. However, in the rightmost panel with lowest availability (θ_S_ = 0.6), the mean number of eye movements made by the model over the range of set size (not counting the initial fixation) goes from 1.9 to 7.6 for positive trials, and 2.9 to 15.0 for negative trials.

At intermediate availabilities (middle panels), the predicted RTs are somewhat negatively accelerated, reflecting how shapes become available for more objects in a single fixation because the objects tend to be closer together with greater set size. A good fit to the RTs (*r*^2^ = 0.98) appears in the third panel where θ_S_ = 0.4, and the predicted RTs are only slightly more negatively accelerated than the observed. Here the range in mean number of eye movements is 1.5–4.0 for positive trials, and 2.4–9.1 for negative trials.

In the visual search literature, much is made from the linearity of the RTs with set size and how the ratio between negative and positive RT slopes is often in the vicinity of 2:1, consistent with a serial self-terminating search (which the Basic Search strategy implements). However, over the availability parameter range shown in Fig. 7, the predicted ratios are {594, 9.8, 2.7, 2.1}. Thus, the RTs are linear and the slope ratio is about 2:1 only to the extent that fixations cover single objects. These results are consistent with a point made by Hulleman and Olivers (2017) concerning the FVF: if the relevant visual property is available over a wide enough area that more than one object can be recognized at time, the search will be faster simply because fewer eye movements are needed to complete the task.

Thus, the RT slopes are a function not only of the time that each eye movement requires; they also depend on *how often* eye movements need to be made. The argument that small slopes imply eye movements are not involved simply fails to hold.

#### Step 2. Add action slips to produce false-alarm errors

The predicted ER results were not shown in Fig. 7 because the initial model predicts zero errors, which is obviously a serious misfit. The model never makes an error because in no case does the visual system produce an *incorrect* representation—the Shape is either veridically available for an object, or it is missing (a *blank* property value), and the search continues until the target is detected or all objects appear to be distractors.

The second step in the explanatory sequence is to modify the initial model to include the action-slip mechanism with the *SlipER* parameter set simply to the overall average ER for false alarms of 0.014. Thus, after the strategy determines the response, the *opposite* response is made with probability *SlipER*. Step 2 improves the ER fit substantially (*aare* goes from 100% to 32%), but miss ER is predicted to be the same as false-alarm ER; the trend of miss ER increasing with set size is not captured, so the *r*^2^ for ER remains at 0.0.

#### Step 3. Include crowding to fit how miss errors increase with set Size

Rather than simply postulating that miss ER increases with “task difficulty” due to some ad hoc error mechanism, we seek to explain why this is so in terms of the visual and strategy mechanisms in the model. As discussed above, the crowding mechanism can produce illusory percepts; these are explored in Step 3 of the explanatory sequence as a source of errors produced by the search strategy.

**Fig. 8:**
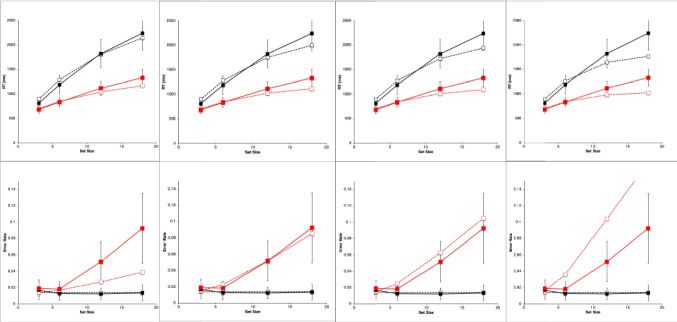
Shape task: Increasing crowding probability increases miss errors in Basic Search. Parameters: θ_S_ = 0.4. Left to right: ϕ_S_ = {0.025, 0.075, 0.1, 0.2}. RT (upper panels, scale 0–2,500 ms) and ER (lower panels, scale 0–0.15). (Color figure online)

Here the crowding mechanism scrambles the Shape property of objects that crowd each other, with a blank (unknown) property value participating in the scrambling. The strength of the crowding effect is specified by the crowding probability parameter ϕ. Accordingly, Fig. 8 expands upon Steps 1 and 2 to show the effects on RTs and ERs of varying the crowding probability parameter, holding availability at the previous well-fitting value θ_S_ = 0.4, with the same Basic Search strategy and *SlipER* remaining at 0.014. The four columns of graphs from left to right correspond to increasing crowding probability, with ϕ_S_ = {0.025, 0.075. 0.1, 0.2}; RT is shown in the upper panels, ER in the lower panels.

Miss ER systematically increases with set size as ϕ_S_ increases (not shown here is that the magnitude of this effect depends on θ_S_). Thus, the availability and crowding mechanisms jointly govern both the RT and ER. A moderately good fit for both RT and ER is obtained with the previous value θ_S_ = 0.4 and ϕ_S_ = 0.075 (center left).

In Fig. 8, the effect of crowding on RT and ER is somewhat subtle and requires careful explanation. A first point is that crowding scrambling cannot produce errors on negative trials because there is no target Shape property on the display (recall that the Shape property is treated as unitary). Thus, as a result, crowding scrambling will not produce an illusory target and a consequent false alarm on a negative trial. So crowding will not affect ER on negative trials.

However, on positive trials, the situation is more complex. The target Shape property is present on the display, but scrambling might move it to a distractor object, producing an illusory target, which would still result in a correct response. Yet as additional fixations are made, it is possible that at some time, *all* objects receive a scrambled *distractor* Shape, including the target object, which thus becomes an illusory distractor. In this case, the Basic Search strategy will immediately halt the trial with an absent response, which is a miss error on a positive trial. The likelihood that the target’s property will be overwritten by a distractor property value depends on both the availability and the crowding probability.

**Fig. 9:**
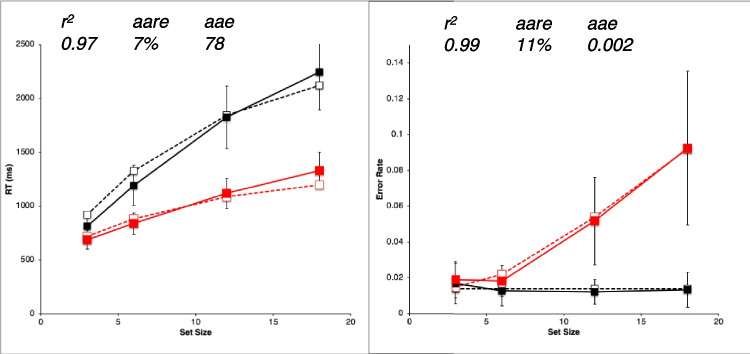
Good fit for Shape task with Basic Search strategy, θ_S_ = 0.425, ϕ_S_ = 0.075, SlipER =.014. (Color figure online)

Figure 9 shows a refined fit, with θ_S_ = 0.425 and ϕ_S_ = 0.075. As mentioned above, some preference was given to obtaining a good fit to ER, since this is a novel goal. Although the predicted RTs tend to be more curvilinear than the data, especially on negative trials, the fits to both RT and ER are an extremely good result considering that only two parameters were adjusted—namely, the availability threshold coefficient and the crowding probability.

#### Why strategy was not changed to fit miss errors

The above crowding account of miss ER differs substantially from the common explanation for miss errors based on some kind of time-out process (cf. Hulleman & Olivers, 2017). According that explanation, if the target has not been detected after some time, or number of fixations, then the participant simply responds absent; this strategy possibility is the limited-fixations option described above in Fig. 6.

However, according to our explanatory-sequence priority order, if an effect can be explained with known perceptual mechanisms (i.e., availability and crowding), then a strategy variation explanation should not be entertained. Moreover, some subsidiary exploration revealed multiple issues involved in trying fit the miss ER data with a limited-fixations strategy instead of crowding. Namely, a different limit is needed for at least three of the set size levels, requiring more parameters than does the crowding mechanism. In addition, the strategy would need elaboration to set the stopping criterion depending on set size, which might require additional visual mechanisms for perceiving numerosity. Thus, relying on crowding to explain miss ER not only conforms to our priority order but also produces a simpler model than would a strategy explanation.

#### Final model for the Shape task

The explanatory sequence for the Shape task, which includes three steps, started with the basic search strategy, eye movements and manual responses. Given these mechanisms, we first found a value for the visual availability parameter that yielded an approximate RT fit. Secondly, we added slip errors, and thirdly, crowding. Parameter values were adjusted at each step to obtain an ultimate very good final fit for both the observed RT and ER data as shown in Fig. 9. The predicted effects reflect only contributions from a “fastest reasonable” strategy and independently documented perceptual-motor limitations. No concept of a perceptual or central processing bottleneck such as covert attention is required to account for data from this prototypical simple visual search task.

### Explanatory sequence of models for the Color task

The next explanatory sequence focuses on Wolfe et al.’s (2010) aggregate data from the Color task. This is a good next choice for explanation because the Color task, like the Shape task, involves a single visual property of the displayed objects. However, unlike in the Shape task, the RTs for the Color task are very flat, fast, and equal for both positive and negative trials. Indeed, Color has often been described as “popping out” in simple visual-search tasks, and it supposedly can be found by a parallel rather than serial search process (cf. Treisman & Gelade, 1980). However, the ER for misses is both relatively constant and higher than the ER for false alarms, which suggests that the “pop out” does not always occur.

Our explanatory sequence for the Color task shows that the “pop out” phenomenon does not stem from a special mechanism. Instead, according to EPIC, *all* the available visual properties are processed in parallel. The flat RTs associated with supposed “pop out” occur simply because the Color target property is highly available over much of the visual field, and the search strategy can simply respond almost immediately to its presence or absence. As mentioned above, the explanatory sequence for this task will just be summarized here. It starts with the same approach as for the Shape task.

**Fig. 10:**
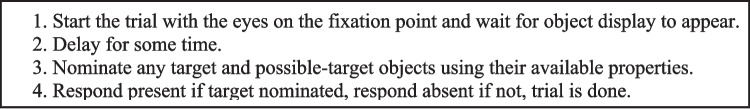
Pseudocode for the Fixed-Eye visual-search strategy

- **Step 1**. Use the Basic Search strategy with slip errors and vary availability to produce flat RTs. Using this strategy, the only way to get equally fast and flat RTs for both response polarities is to have the Color property be extremely available, resulting in no eye movements being necessary for either positive or negative responses even at the largest set size.
- **Step 2**. Crowding fails to account for misses. Unlike in the Shape task, introducing crowding scrambling has essentially no effect on ER, even at high values of ϕ_C_. This happens because the high Color availability means that eye movements are rarely made, so scrambling rarely happens more than once (when the display first appears), so extremely few errors occur from crowding. There are no visual parameter settings that reproduce both the RT and ER results.
- **Step 3**. Use a fixed-eye strategy and vary availability to fit flat RTs and misses. The third step in priority order is to try a strategy that will always produce flat RTs—namely, the Fixed-Eye Strategy shown in Fig. 10. Here the eyes are kept on the central fixation point, and after a single nomination phase, the response is chosen. If a target has been nominated, a target-present response is made. If not, the target-absent response is made. This strategy essentially corresponds to the common instructions in visual search experiments that call for keeping the eyes on the fixation point. Because of extensive practice, participants might discover that they can almost always detect the object colors without moving their eyes and so adhere to the task instructions.

Thus, on a positive trial, if the target Color is available from the fixation point, the chosen response will be “present”. If the target Color is not available, the chosen response will be “absent”, producing some misses. On a negative trial, the intended response will be “absent.”

Of course, all responses might be switched to the opposite ones by slip errors at the constant *SlipER* rate. Thus, if the Color property is not always available, miss responses will be more frequent than false alarms, and the ERs are determined only by θ_C_ and *SlipER*. Concomitantly, RTs are constant, determined only by the fixed time required for a simple process of perception, decision, and response.

#### Final model for the Color task

**Fig. 11:**
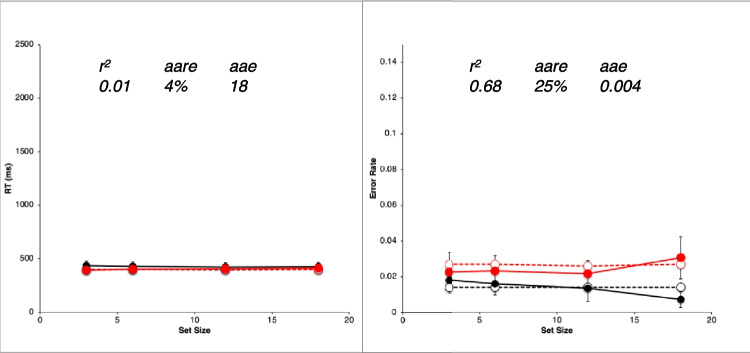
Color task: Good fit with Fixed-Eye strategy, θ_C_ = 0.11, SlipER = 0.014. (Color figure online)

Figure 11 shows the fit for the final model with θ_C_ = 0.11, ϕ_C_ = 0.0, and SlipER = 0.014. Because the RTs and ERs are flat across set sizes, the *r*^2^ values are only zero and 0.68 respectively, but the other goodness-of-fit measures are very good.

Effects observed in the Color task can be explained by the extremely simple Fixed-Eye strategy and the availability mechanism. No special “pop-out” mechanism is required. The Color property is sufficiently available that eye movements are rarely needed and can be eliminated by the Fixed-Eye strategy if just a few more misses are accepted.

An effect of crowding was not needed to adequately fit these data. However, a crowding-probability parameter value could be assigned to the Color property without affecting the goodness-of-fit for the final Color-task model. In fact, the next explanatory sequence shows how crowding plays a key role in the Conjunction task, even though both Color and Orientation are highly available.

### Explanatory sequence of models for the Conjunction task

Modeling the Conjunction task confronts an interesting challenge, given that this dataset has a pattern of effects that are different from the two extremes of the Shape and Color tasks. Unlike in the Color task with its flat and short RTs (Fig. 11) caused by the absence of eye movements, the Conjunction-task RTs have substantial slopes. However, they are not as steep as in the Shape task (Fig. 9), which required many eye movements.

Furthermore, unlike in the Color task—where miss ERs were flat with set size and crowding played a negligible role due to the lack of eye movements—the Conjunction-task miss ERs increase substantially with set size, but to a lesser extent than for the Shape task, in which crowding produced many misses.

Successfully explaining these effects is an important contribution of present modeling work. The difference between performance on single-feature and conjunctive search tasks was a key phenomenon that originally motivated covert-attention shifting theories of visual search (cf. Treisman & Gelade, 1980). So our present challenge is to satisfactorily explain this difference without including a covert attention mechanism. The following summarized explanatory sequence shows how this can be done.

- **Step 1**. Use basic search strategy, slip errors, and vary availability to fit RT slopes. As for the other tasks, the initial model for this task assumes the Basic Search strategy. As explained above, the Conjunction task requires more complicated nomination rules for targets and possible targets, because there are different possible combinations of the two properties of Color and Orientation, which are assumed to be independently available.
- In Step 1 of our explanatory sequence for the Conjunction task, we assume simply that the availability parameter θ_C_ for Color has the same value as in the best fit for the Color task, namely θ_C_ = 0.11, and it will be held constant at that value in what follows. Unfortunately, the Wolfe et al. (2010) dataset did not include an Orientation single feature task that could be used to separately estimate θ_O_ for stimuli of the same size and shape. However, from the available literature, it appears that Orientation is less available than Color. Step 1 of this explanatory sequence revealed that a good fit to the RTs occurs with an Orientation availability parameter θ_O_ = 0.225 or 0.25, consistent with Orientation being less available than Color.
- **Step 2**. Vary crowding to fit miss errors. Since crowding explained the miss ER in the Shape task, adding crowding effects seems like a good second step to fit the miss ER in the Conjunction task. However, a severe problem immediately appears. With *SlipER* set to the observed very low false-alarm ER of 0.014, even small crowding probabilities (e.g., ϕ_C_ = ϕ_O_ = 0.025) produce a *massive* predicted false-alarm ER that greatly exceeds the observed value and increases steeply with set size.
- This misfit results from the combination of crowding and the Basic Search strategy as follows: The Conjunction task has almost equal numbers of Red and Green property values on the display; there are also almost equal numbers of Horizontal and Vertical property values. Because Color is very available, and Orientation moderately available, many instances of values for these properties are available at the same time. Consequently, as described previously, crowding effects can cause illusory targets and illusory distractors when the Color and Orientation property values get scrambled between the objects. If a Vertical value replaces a Horizontal value on a Red distractor, or a Red value replaces a Green value on a Vertical distractor, the result is an illusory target.
- Furthermore, the Basic Search strategy will terminate as soon as an object appears to be a target regardless of whether it has been fixated. Consequently, on negative trials, even with low crowding probabilities, illusory targets are common enough to cause the strategy to frequently terminate early with a false alarm. Also, the false-alarm rate increases steeply with set size, as more objects crowd each other on the display.
- Thus, this model is *remarkably incorrect* even though it incorporates all the previously considered visual and motor mechanisms and parameters. The implication is that performance in the Conjunction task involves complexities beyond the previous models for the Shape and Color tasks. Following the preferred priority order for adding or changing mechanisms, a change of the task strategy is the next step in the explanatory sequence for dealing with these complexities.
- **Step 3**. Use confirm-positive Basic Search and vary crowding to fit RT and ER. The false alarms can be prevented with a *confirm-positive* variation of the Basic Search strategy. If the target appears to be present, it is double-checked. If the apparent target object is already fixated (eccentricity ≤1°), a present response is made because crowding should be insignificant; if not, the strategy moves the eyes to the apparent target, which would alleviate any crowding. The strategy responds present if this object is indeed the target or continues the search if not. This confirmation takes extra time, both for the additional eye movement and production-rule cycles.
- The strategy is a simple and well-specified form of “double-checking” sometimes proposed in visual search models (cf. Hulleman & Olivers, 2017). Unfortunately, because double-checking could go on for too long, it produces steeply sloped negative-trial RTs; there is no combination of availability and crowding parameters that will fit both the RT and ER data.
- **Step 4**. Basic Search with confirm-positive and limited-fixations for RTs and misses. Due to these considerations, the task strategy was modified to include the limited-fixations option (Fig. 6) of responding absent after a specified number of fixation eye movements *Nmax* = 3. This modification produces a fairly good fit to both RTs and ERs.
- **Step 5**. Use confirm-both strategy to fit positive RTs. Unfortunately, a small systematic deviation still remains after Step 4; predicted RTs for negative trials are systematically somewhat too fast, at the smallest set size. This happens because a target-present response often requires an extra confirmatory eye movement, but a target-absent response does not.

**Fig. 12:**
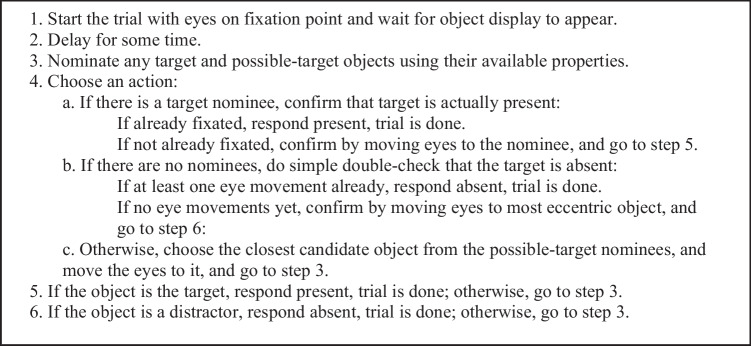
Pseudocode for the Basic Search with confirm-both strategy. Each execution of Steps 3 through 6 requires one 50-ms cycle. Steps 4a, 4b, and 4c can also require manual and eye-movement time

Consequently, in Step 5, a very simple form of confirm-negative double checking was added to produce the confirm-both variation of Basic Search, shown in Fig. 12. Here, the confirm-positive check described above is done in strategy Step 4a. The confirm-negative check is done in strategy Step 4b: before responding negative at the beginning of the trial, check that the current most eccentric object is a distractor. The limited-fixations option (Fig. 6) is applied before each eye-movement is commanded.

#### Final model for the Conjunction task

**Fig. 13:**
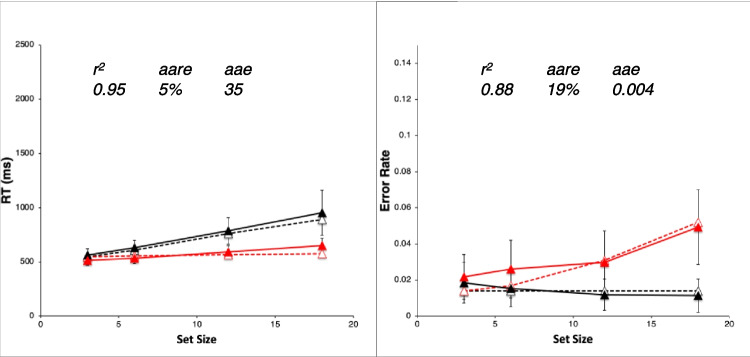
Good fit for the Conjunction task using the confirm-both strategy with limited-fixations. θ_C_ = 0.11, ϕ_C_ = 0.025, θ_O_ = 0.2, ϕ_O_ = 0.025, SlipER = 0.014, N_*max*_ = 3. (Color figure online)

The final model whose fit appears in Fig. 13 reveals that participants performed the Conjunction task *very differently* than the Shape and Color tasks. Even when the probability of crowding is very low, the scrambling of highly available Color and Orientation properties between perceived objects produces many illusory targets and illusory distractors, far more than occur in the Color and Shape tasks. Double-checking a tentative positive response on negative trials could take a very long time, so participants apparently limited their fixations in order to shorten RTs at the expense of suffering some Misses. Furthermore, illusory distractors apparently required an occasional double-check before a negative response.

As discussed previously, differences between observed performance for the Conjunction task versus the Color task inspired Treisman and Gelade (1980) to propose their theory that serial covert-attention shifting is required for binding features into perceived objects. However, the present explanatory sequences of EPIC models show that performance of the Conjunction task differs from performance of the Color task because of two aspects fundamentally different from what Treisman and Gelade (1980) had in mind. First, the early-vision limitations of availability and crowding cause visual percepts during the Conjunction task to be very ambiguous—illusory targets and illusory distractors are common. Second, a more complex eye-movement strategy is required to resolve this ambiguity while producing acceptably fast and accurate responses.

## Model results for all three tasks

### Testing the architecture as well as individual models

A cognitive architecture represents a claim that there is a common set of theoretical components that can account for performance in a variety of tasks. In the present case, these consist of visual perceptual mechanisms, eye and manual movement mechanisms, and a strategy execution mechanism. Tasks that involve different stimuli may require different parameter values in the perceptual mechanisms. Likewise, tasks whose procedural requirements are different will require different strategies; as mentioned above, the choice of strategy is a kind of parameter setting. *The contribution of the architecture is to show how a variety of individual phenomena can be explained by models in the same framework of mechanisms and parameters.* Thus, a useful question is how well did EPIC models succeed across all the tasks?

**Fig. 14:**
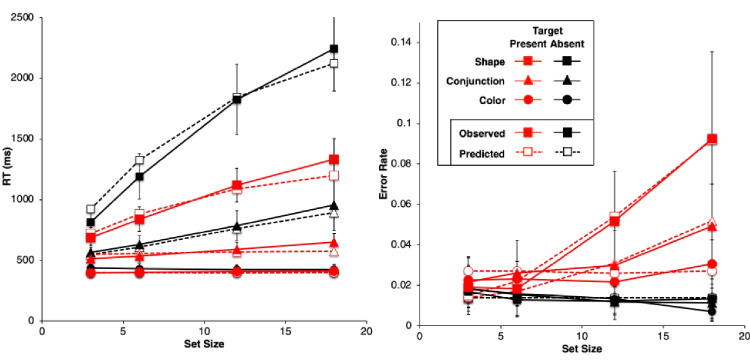
Excellent fit to the aggregated data using the models for each task. Predicted (open points, dotted lines) and observed (solid points and lines) correct trial RT (left panel) and ER (right panel). Shape: squares, Color: circles, Conjunction: triangles; positive trials: red, negative trials: black. The 95% confidence intervals are based on the standard error of the mean of the subjects’ mean values underlying each data point and thus reflect between-subject variability. (Color figure online

To help answer this question, Fig. 14 shows predicted RTs and ERs compared with the data for all three tasks. Table 2 shows the parameter values used in the final models for all three tasks. Also shown in Table 2 are the goodness-of-fit metrics for RTs and ERs from each individual task condition, together with the averages of these metrics across task conditions. The separate and average fits are all excellent. When the metrics are computed for the whole set of 24 RT data points, the very high *r*^2^ values show that the models predict the mixture of flat and differently sloped RTs very accurately. Furthermore, the fit to the ERs is also excellent in terms of all metrics, which is a new result for models of simple visual search.

**Table 2:** Model parameters and goodness-of-fit metrics for all task data

		Shape/Color		Orientation				RT			ER		
Task	Strategy	θ	ϕ	θ	ϕ	*N*_*max*_	*SlipER*	*r*^2^	*aare*	*aae*	*r*^2^	*aare*	*aae*
Shape	Basic Search	0.425	0.075				0.014	0.97	7%	79	0.99	11%	0.002
Color	Fixed-eye	0.11					0.014	0.01	4%	18	0.68	25%	0.004
Conjunction	Basic Limited Confirm-both	0.11	0.025	0.20	0.025	3	0.014	0.95	5%	35	0.88	19%	0.004
Average* of separate fit metrics for each task								0.96	5%	44	0.85	18%	0.003
Fit metrics using all data points from the three tasks								0.98	5%	44	0.95	19%	0.003

* Averages of *r*^2^ for RT do not include near-zero value for Color task

In short, we started with the dataset shown Fig. 2 and now have a set of models that almost perfectly predict these data by using a common set of EPIC architectural mechanisms that does not include covert-attention shifting.

Yet the confidence intervals around the observed means in Fig. 14 are quite large for some of the RT data, and especially so for the miss ER data. This occurred because the mean RTs and also ERs from individual participants differ substantially. For example, in performing the Shape task, some participants achieved almost error-free performance, while one participant produced 23% errors at set size 18. Different patterns of RT effects also occurred across individual participants in the same task condition. To address these concerns, the next section considers further EPIC models that focus on explaining such individual differences.

## Modeling search performance for individual participants

Like for many topics in human cognition and performance, there has been relatively little consideration of individual differences in visual search, even though such differences surely occur regularly. For example, major individual differences have appeared in some published data from simple visual-search tasks (e.g., Wolfe et al., 1989). Furthermore, despite our success with modeling the aggregated data from Wolfe et al. (2010), the previously presented explanatory sequences show that both different parameter values and different task strategies can lead to large differences in predicted performance.

We are therefore concerned that ignoring systematic individual differences might yield misleading theoretical conclusions based on the present research. Accordingly, the next subsections explore these possibilities further. To do so, predicted results from explicit EPIC models of individual differences in the Wolfe et al. (2010) data are introduced.

## Representing individual differences in EPIC models

The EPIC architecture provides a straightforward theoretical framework for characterizing individual differences in terms of two sources:

1. Different parameter values of some architectural mechanism(s): For example, participants certainly differ in their visual characteristics that may affect search performance (e.g., Verissimo et al., 2021). Unfortunately, Wolfe et al. (2010) did not measure parameters like visual availability for their participants, so there is no direct way to assess this source of individual differences. Nevertheless, it seems likely that satisfactory models for individual participants might require individual parameter values for best fits to the data of Wolfe et al. (2010)
2. Different strategies for performing the same task also may be a source of systematic individual differences: For example, participants might differ in which variant of the Basic Search strategy they used to perform the Conjunction task. Substantial strong prior evidence exists to support this conjecture. Past EPIC-inspired experimental work has shown that strategy differences between individuals can and do produce misleading factor effects in aggregate human-performance data (e.g., Schumacher et al., 2001; Thompson et al., 2015).

In principle, averaging over participants who have different perceptual/motor parameters and/or different task strategies could yield average data substantially different from what every single participant produced (cf. Newell 1973, pp. 294–296; Reitman, 1970). As a result, the models for the aggregate data would not correspond to the performance of any individual participants. Thus, it is worth examining whether systematic interpretable individual differences occurred in Wolfe et al.’s (2010) dataset, and whether EPIC models can account for them as well as the aggregated average data.

## Present approach to modeling individual differences

A straightforward approach to achieve our objective for understanding individual differences in simple visual search would be to construct a separate best-fitting model for each of Wolfe et al.’s (2010) participants. However, given Wolfe et al.’s experimental design, this would yield a total of 28 separate models. To make sense of the results, we would need some way of characterizing how these 28 models are both similar to and different from each other, such as grouping the individual models in some way.

Because of these concerns, we instead took a more economical approach to arrive at a set of models with clear similarities and differences: This involved first identifying *clusters* of participants in each task condition. The individual participants in each cluster had mean RTs and ERs that were similar in magnitude and pattern of factor effects. We then constructed a good-fitting model for each cluster’s mean data, which manifested a particular combination of parameter values and task strategy

The following sections show that for the most part—despite a few noteworthy exceptions—the parameters and strategies needed to model the aggregate data of Wolfe et al. (2010) also sufficed to account for data from each of the clusters of individual participants.

## Cluster analysis

To get a first look on whether there were meaningful systematic individual differences, we plotted mean RT and ER graphs for each participant. On informal inspection, these graphs seemed to fall into a small set of patterns for each task condition. This impression was formally confirmed through a cluster analysis of the participants’ data in each task condition.

Of course, cluster analysis is usually done on very large datasets. Its application here to a small dataset—only nine or 10 participants in each task condition—simply provided a way to formalize what would otherwise be a purely intuitive “eyeballing” process. However, there were about 500 trials per data point per participant, so the individual-participant mean data were highly reliable, which somewhat mitigates the overall small sample size.

The RT variables used to identify the clusters were the intercept, slope, and best-fit quadratic coefficients for RTs from positive and negative trials. The accompanying ER variables were mean values for false alarm and miss ER, and the maximum miss ER at set size 18. Using R package *cluster* (Maechler et al., 2017), good solutions were obtained with three clusters in each task condition (see Kieras, 2020, for details). The final participants in the clusters were chosen so that averaging the RT and ER data within a cluster preserved the basic qualitative and quantitative trends for the individual participants in the cluster. This required moving two participants out of the computed clusters into clusters of one participant each. We did not fit models to these single-participant clusters. The RT and ER data in each remaining multiparticipant cluster were averaged to produce the mean data for that cluster. Finally, a model was fit to the mean data in each multiparticipant cluster, using the same explanatory-sequence principles already described.

For brevity, only the mean data from each multiparticipant cluster and corresponding final model fits are presented here. Details can be found in Kieras (2020). In the following subsections, each cluster of participants is labeled with a short description that summarizes their RT and ER characteristics, with a left-to-right order based on increasing ER.

## Shape-task clusters of participants

**Fig. 15:**
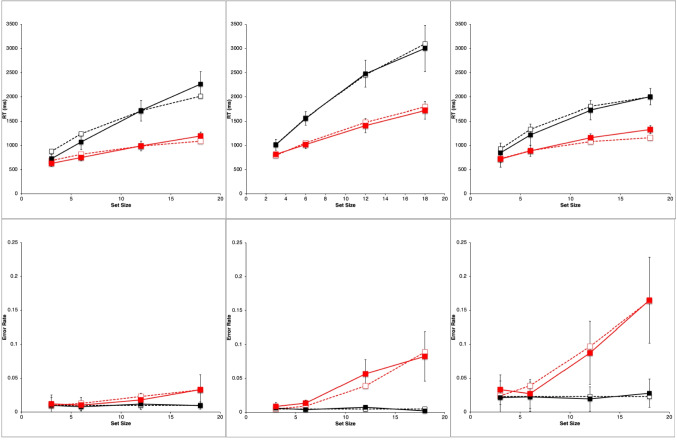
Mean observed data and model predictions for the Shape participant clusters: Observed (solid lines and points) versus Predicted (dotted lines, open points). Upper panels: RT, scale: 0–3,500 ms. Lower panels: ER, scale 0–0.25. Clusters: Left panels: Slow/LowER. Middle panels: VerySlow/MedER. Right panels: NegAcc/HighER. The 95% confidence intervals on each observed data point are based on the mean RT and ER for that data point for each of the 2–3 participants included in the cluster

Figure 15 shows the observed mean RTs and ERs (solid points and lines) for the three modeled participant clusters in the Shape task. Interestingly, the confidence intervals surrounding the data points in each cluster are relatively narrow compared to those in the overall aggregate data for the Shape task (Fig. 2). Thus, even though only a few participants are being averaged together here, the participants in each cluster are similar enough that their mean data tend to be less variable than their aggregated data.

An EPIC model was fit to the mean RT and ER data for each Shape cluster with *SlipER* set to the mean false-alarm ER for that cluster. The obtained model fits are shown in Fig. 15 (open points and dotted lines). Their parameters and goodness-of-fit statistics appear in Table 3.

**Table 3:** Model parameters and goodness-of-fit metrics for the Shape-task clusters

Cluster & Strategy	Shape		*SlipER*	RT			ER		
	θ	ϕ		*r*^2^	*aare*	*aae*	*r*^*2*^	*aare*	*aae*
Slow/LowER *Basic Search*	0.375	0.025	0.01	0.96	9%	101	0.92	15%	0.002
VerySlow/MedER *Basic Search*	0.6	0.05	0.005	1.00	3%	41	0.94	43%	0.005
NegAcc/HighER *Basic Search*	0.435	0.150	0.023	0.96	6%	67	0.98	16%	0.005
Average fit metrics				0.97	6%	70	0.94	25%	0.004

All three Shape-task clusters differ from each other in terms of their visual parameters and *SlipER*. However, the models for them use the same Basic Search strategy with unlimited eye fixations that worked for the aggregate Shape-task data. Thus, in this case, the clustered data differ from the aggregate data because of large differences in the individual parameter values rather than different individual task strategies.

## Color-task clusters of participants

**Fig. 16:**
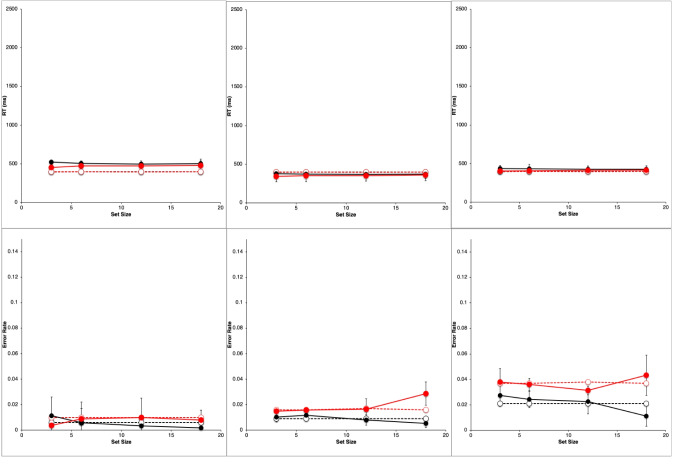
Mean observed data and model predictions for the Color-task clusters. Observed (solid lines and points) versus Predicted (dotted lines, open points). Upper panels: RT, Scale: 0–2,500 ms. Lower panels: ER. Scale: 0–0.15. Left panels: Slow/LowER. Middle panels: Fast/MedER. Right panels: Fast/HighER. The 95% confidence intervals on each observed data point are based on the mean RT and ER for that data point for each of the 2–4 participants included in the cluster. (Color figure online)

Figure 16 shows the observed mean RTs and ERs (solid points and lines) for the three participant clusters in the Color task. As before for the Shape task, the confidence intervals surrounding the data points in each cluster are relatively narrow. The slope ratios are not meaningful because the positive and negative RT slopes are very small and noisy.

A good model fit was obtained for each of the three participant clusters in the Color task (Fig. 16, open points and dotted lines). These fits involved the Fixed-eye strategy, *SlipER* set to the false-alarm ER for each cluster, and the availability parameter θ_C_ adjusted to fit the miss ER for each cluster. Because of reasons discussed above regarding the aggregate-data model for the Color task, the Color crowding-probability parameter ϕ_C_ was set to a place-holder value of 0.025, consistent with the models for the Conjunction task.

As also shown in Table 4, the fits for the participant clusters in the Color task appear to be very good, given that the *r*^2^ metric is not meaningful when both observed and predicted RTs are essentially constant with set size.

**Table 4:** Model parameters and goodness-of-fit metrics for the Color-task clusters

Cluster & Strategy	Color			RT			ER		
	θ	ϕ	*SlipER*	*r*^2^	*aare*	*aae*	*r*^2^	*aare*	*aae*
Slow/LowER* Fixed-eye*	0.09	0.025	0.006	0.06	18%	89	0.10	71%	0.003
Fast/MedER *Fixed-eye*	0.10	0.025	0.009	0.10	11%	38	0.59	22%	0.003
Fast/HighER *Fixed-eye*	0.115	0.025	0.021	0.12	5%	22	0.69	21%	0.004
Average fit metrics				0.09	11%	50	0.46	38%	0.003

The account for the Color task clusters is very simple: As in models for the overall aggregate data from the Color task, participants in each of the underlying clusters apparently used the Fixed-Eye strategy. This made the RTs flat and fast, while crowding had a negligible effect. Differences in *SlipER* and Color availability produced distinct levels of miss and false-alarm ER.

## Conjunction-task clusters of participants

**Fig. 17:**
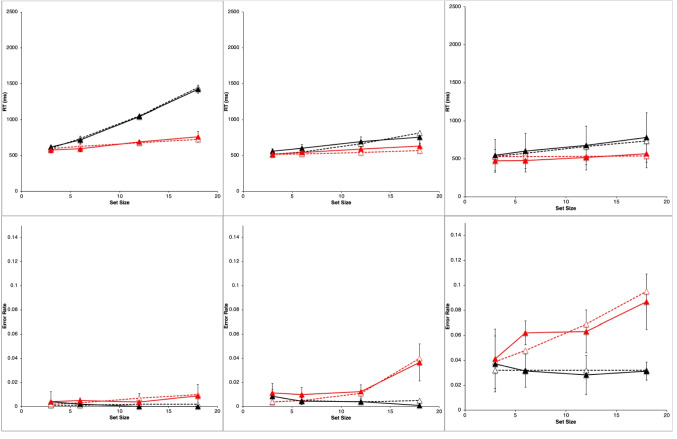
Mean and observed data and model predictions for the Conjunction participant clusters: Observed (solid lines and points) versis Predicted (dotted lines, open points). Upper panels: RT, scale 0–2,500 ms. Lower panels: ER, scale 0–0.15. Clusters: Left panels: Sloped/LowER. Middle panels: AlmostFlat/MedER. Right panels: AlmostFlat/HighER. The 95% confidence intervals on each plotted point are based on the mean RT and ER for that data point for each of the 2–4 subjects included in the cluster. (Color figure online)

Figure 17 shows observed mean RTs and ERs (solid points and lines) for the participant clusters in the Conjunction task. The first cluster (Sloped/LowER; left panels) is very different from the other clusters. It has steeply sloped mean RTs for negative trials, and extremely low mean ERs. The second and third clusters have very similar and less sloped mean RTs, but very different patterns of mean ERs. As the relatively large confidence intervals reveal, the third cluster (AlmostFlat/HighER; right panels) is more heterogenous than the other two clusters.

The final model fits also appear in Fig. 17 (open points and dotted lines) for the participant clusters in the Conjunction task. The associated model parameters and goodness-of-fit metrics appear in Table 5. While the Basic Search strategy with the confirm-both option produced the best fit for all three of these clusters, they differed in terms of how many eye movements were allowed before the search terminated with a negative response. For the first cluster (Sloped/LowER; left panels), the number of fixations was not limited. These participants apparently chose to minimize ER at the expense of long and highly sloped RTs. Participants in the second and third clusters (Fig. 17; middle and right panels) chose to produce almost-flat and faster RTs at the expense of more errors.

**Table 5:** Model parameters and goodness-of-fit metrics for Conjunction-task clusters

*Cluster & Strategy*	Color		Orientation				RT			ER		
	θ	ϕ	θ	ϕ	*N*_*max*_	*SlipER*	*r*^2^	*aare*	*aae*	*r*^2^	*aare*	*aae*
Sloped/LowER *Basic Unlimited* *Confirm-both*	0.15	0.025	0.25	0.05		0.0015	0.99	3%	21	0.51	53%	0.002
AlmostFlat/MedER *Basic Limited* *Confirm-both*	0.10	0.075	0.15	0.075	3	0.0045	0.90	6%	40	0.90	76%	0.003
AlmostFlat/HighER *Basic Limited* *Confirm-both*	0.11	0.025	0.18	0.025	2	0.032	0.91	6%	32	0.91	10%	0.005
Average fit metrics							0.93	5%	31	0.77	46%	0.003

Taken together, these results reveal that the aggregated data hid a major difference between individual participant strategies for the Conjunction task. Apparently, the Sloped/LowER participants (Fig. 17; left panels) rose to the challenge of minimizing errors by not limiting their eye fixations, despite this requiring much longer RTs. In contrast, the AlmostFlat participants (Fig. 17; middle and right panels)—faced with 4,000 trials of this tricky task—opted to “get it done” even at the expense of more errors.

Importantly, on balance, the Conjunction-task participant clusters therefore illustrate the perils in modeling aggregated data. Such perils may prevail especially when—as in the study by Wolfe et al. (2010)—a lack of clear instructions and incentives allows participants to choose their task strategies rather freely. This freedom might be significant particularly in the Conjunction task, where the perceptual ambiguity of the stimulus displays requires a much more complex strategy than do the other tasks. Under these challenging conditions, it is unsurprising that individual participants would be more varied in their specific strategies.

## Overall summary of results: Excellent model fits to aggregated and clustered data

Table 6 summarizes the goodness-of-fit metrics for all the final models discussed in previous sections, combining the averages from Table 2 (aggregated data), Table 3 (Shape-task clusters), Table 4 (Color-task clusters), and Table 5 (Conjunction-task clusters). The task strategies and perceptual-motor parameter values in these models produced a set of excellent model fits to both observed mean RTs and ERs for the aggregated data from each of the three task conditions. Furthermore, the model fits were excellent for the clusters of individual participants whose data contributed to each condition, even though each cluster contained at most four people.

**Table 6:** Summary of goodness-of-fit for aggregated and cluster data

Source of model fit metrics	RT			ER		
	*r*^2^	*aare*	*aae*	*r*^2^	*aare*	*aae*
Average* of separate fits of each task with aggregated data	0.96	5%	44	0.85	18%	0.003
Overall fit of all aggregated data (all 48 mean data points)	0.98	5%	44	0.95	19%	0.003
Average* of separate cluster fits for all three tasks	0.95	7%	50	0.72	36%	0.003

* Averages of *r*^2^ for RT do not include near-zero values for Color tasks

The major difference between the models for the various tasks is the strategy required to perform the visual search given the perceptual properties of the stimulus displays. For the Shape task, a lengthy and systematic search with lots of fixations was needed because the relevant perceptual property is not very available. In contrast, a fast fixed-eye strategy sufficed for the Color task, because the relevant perceptual property was extremely available. Furthermore, most elaborately, various versions of a double-checked and time-out strategy were necessary for the Conjunction task to deal with how visual crowding produces many illusory combinations of the color and orientation properties.

The fact that most participants in a task chose the same strategy may be explained by the large amount of practice that they had with the task. Although performance incentives in the study by Wolfe et al. (2010) were not explicit, apparently the need to complete a very large number of trials with reasonable response accuracy was enough to encourage most participants to converge on strategies that shared the “fastest reasonable” property of the model strategies. Perhaps similarly stable results with fewer trials would have occurred if clear and unambiguous performance incentives had been used.

## General discussion

### Future challenges for EPIC models of visual search

#### Viability of the EPIC approach

An advantage of EPIC as a theoretical framework is that it allows a mixture of modeling approaches—the architecture accommodates traditional mathematical models for perceptual and motor mechanisms along with symbolic “program-like” procedural representations for task strategies as production rules. This division of labor is pragmatic: writing production rules for mathematical functions is extremely difficult, while trying to represent strategies in conventional mathematics is clumsy. Future work applying EPIC to a wider variety of visual search tasks may determine whether its pragmatism continues to be viable.

#### Other types of search tasks

As mentioned previously (see Basic EPIC Mechanisms section), EPIC models have been proposed to account for performance in various complex search tasks. These models involve predicting the times required to select computer menu items, as well as the times required to find multiply cued target objects in very large numbers of display objects. In contrast, the present article has focused strictly on simple visual-search tasks that require present/absent responses for displays that contain moderate numbers of easily perceived simple objects in relatively large spatial areas.

Furthermore, there is also another literature on visual search tasks where, in response to a cue such as a color, participants must find a matching target amongst perceptually challenging objects and respond to another feature (e.g., orientation) of it. The main concern there has been to study how the search RT depends on the similarity of the cue information to the objects being searched. The accompanying theoretical framework has assumed that visual processing of the displayed objects occurs in parallel through a race for attentional selection (e.g., Buetti et al., 2016; Bundesen, 1990; Duncan & Humphreys, 1989). EPIC models may also be applicable to such search tasks, but this will require devising early-vision mechanisms and task strategies that are sensitive to visual similarity.

#### Analysis of visual features

As part of their work, Duncan and Humphreys (1989) studied simple visual search of displayed shapes composed of a few line segments (e.g., rotated *L* or *T* shapes) with hypothetical feature structures. They found that the search process was slower when targets had features more similar to distractors, and when distractors were less similar to each other (cf. Lleras et al., 2022). In contrast, our present models do not rely on an analysis of features; rather the overall availability parameter of a unitary Shape property is estimated to fit the data. EPIC as a cognitive architecture does not assume any particular feature analysis of visual stimuli; it only provides a framework for features as a property-value representation that can be processed by production-rule task strategies. A further challenge will be to show in detail that EPIC models can elegantly and generally explain findings like those of Duncan and Humphreys (1989).

#### Distractor heterogeneity effects

These effects are a more specific form of the similarity effects already described above, in which it is harder to find the target amid distractors that are unlike each other. This presents a particular challenge for the task strategies in the present EPIC models. With all these strategies, in the absence of a known target, the present models consider possible targets, defined as having an unknown value for a target property. If there are no such objects, an “absent” response is made. The actual nontarget property values are irrelevant for this process, which implies that distractor heterogeneity would have no effect. It is possible that the heterogeneity effects reported by Duncan and Humphreys (1989) might be due to early-vision limitations such as greater crowding. If not, then a rather different search strategy might be required for EPIC models to explain effects of distractor heterogeneity.

#### Negatively accelerated RT functions

The family of visual-search theories in which all items are processed in parallel and have stochastic completion times can predict logarithmic rather than linear RTs with set size (Buetti et al., 2016). However, as shown in our explanatory sequence for the Shape task (see Explanatory Sequence of Models for the Shape Task section), current EPIC models also can produce negatively accelerated RT functions, even though the underlying sensory/perceptual processing times have constant durations. Because the objects are denser at greater set sizes, this might simply reflect more objects having available properties at a given eye location, as in an FVF. Perhaps additional experimental data in which object density is less confounded with set size could be collected and modeled to resolve the source of negatively accelerated RT functions.

#### The meaning of “attention”

The human-performance literature contains multiple alternative ideas about “attention” (Luck & Vecera, 2002), with some recurring doubts cited above (cf. Current Status of Covert-Attention Shifting in Research on Simple Visual Search section) concerning whether it is a useful concept. Interestingly, the present EPIC *task strategies* perform at least some of the functions of “attention” in visual search. For example, the strategy determines (a) which visual properties of what displayed objects are relevant to choosing the response and (b) to which objects the eyes should be moved for further information. This rather limited concept of attention-as-strategy together with known early-vision limitations suffices to account for the results of Wolfe et al. (2010).

Another future challenge for EPIC models would involve considering a larger range of empirical phenomena to see if any other “attention” functions are required. For example, such phenomena would include the widely accepted, direct top-down cognitive facilitation of visual perception without eye movements, as described by Posner (1980). Currently, EPIC does not have any way for a cognitive strategy to directly influence the perceptual processing of a specific visual property, object, or spatial region. However, it would certainly be possible to add such a mechanism.

For now, though, the question remains: do available data require such complications? Perhaps not. Fixational eye movements (i.e., microsaccades around the fixation point) might account for these spatial attentional-cuing effects (e.g., Engbert & Kliegl, 2003; Rolfs, 2009). This suggests that the facilitation could stem from a type of eye movement rather than direct cognitive enhancement of visual processing.

### Recommendations for future experiments on simple visual search

#### Adopt routine eye tracking

A paramount methodological conclusion follows from the present research. The EPIC models proposed here show that early-vision limitations and eye movements controlled by task strategies can account extremely well for empirical data from experiments on simple visual search. This demonstration conflicts with prior claims by other investigators that early-vision limitations and/or eye movements are irrelevant for understanding simple visual search. Future visual search experiments could further refute (or support) such claims by collecting eye-tracking data along with RT and ER measurements (cf. Hulleman & Olivers, 2017). Since eye tracking has become much less expensive, more precise, and easier to implement than in the past, there are strong reasons for pursuing such an agenda, given prevailing theoretical issues.

#### Measure perceptual parameters of individual participants

To account best for observed empirical data on simple visual search, our explanatory-sequence approach has a well-defined way of constructing successively better-fitting computational models. It gives priority to adjusting assumed values of perceptual parameters before trying various modifications of an assumed task strategy. This heuristic stems from a belief that the mechanisms underlying these parameters are less mutable than strategy choices.

However, we lack adequate information about plausible values for these parameters. Investigators in different laboratories almost never use the same stimuli for experiments on visual search, nor do they usually measure the characteristics of their stimuli in separate psychophysical tasks. Only on rare occasions (e.g., Verissimo et al., 2021) have parameters such as visual availability been measured for individual participants and related to their performance. Faster progress would occur if such data were collected in future work on simple visual search.

#### Provide explicit performance incentive schemes

Together with perceptual mechanisms, task strategies are also major contributors to explaining visual-search performance, both for different search tasks and for different participants in each task. However, as mentioned previously, there has been another weakness in many past experiments on simple visual search. They have typically neglected to provide participants with an explicit incentive scheme that helps clarify the relative importance of achieving short RTs versus low ERs.

This deficiency is quite problematic. If participants are not clearly and strongly incentivized to perform a given task in a particular way, then they are likely to devise their own idiosyncratic strategies, which may confuse efforts to explain how the task is done. For example, in analyses of results from the present Conjunction task, we found there were three participant clusters that apparently differed greatly in their preferences for short RTs versus low ERs. These differences were captured by different task strategies. As mentioned previously, this may have happened because Wolfe et al. (2010) simply instructed participants to “respond as quickly and accurately as possible,” which is an ambiguous and contradictory directive.

Consequently, to avoid such circumstances, future experiments on simple visual search should use incentive schemes (e.g., Sternberg, 2016, Appendix B) that can stabilize strategy choices, thereby helping purify both aggregate and individual participant performance.

### Recommendations for future theorizing about simple visual search

#### Be skeptical about covert attention

Past work with the EPIC cognitive architecture (e.g., Meyer & Kieras, 1997a, b, 1999) and the work in the present paper demonstrate how well human performance can be accounted for by computational models that (1) do not have a priori assumed hypothetical cognitive limitations such as central response selection or attentional bottlenecks, but (2) do include known perceptual/motor constraints and task strategies.

The present models’ success demonstrates that a particular hypothetical central cognitive limitation, such as needing to shift covert attention between spatial locations, is superfluous and likely irrelevant for a correct theoretical account. Consequently, we believe that future research on visual search should endeavor to advance beyond these hypothetical concepts. In fact, our results are sufficiently compelling to make us wonder why covert attention has received so much consideration in past visual search research, while more concrete and essential mechanisms have been largely ignored (cf. Rosenholtz, 2024). This is a question for historical analysis of the origins of covert-attention shifting theories.

#### Use explanatory sequences to construct models

During initial development of the present models for simple visual search, our past work on developing EPIC models provided valuable general guidance. Yet this often seemed like a random walk through the space of possible models. Subsequent progress was accelerated when we began systematically applying the explanatory-sequence approach to constructing further models. It involves a rigorous priority order for adding mechanisms to achieve successively better models, demonstrating that they are both necessary and sufficient to account for the data. The same method could be useful as well for future explorations of human performance with other cognitive architectures and theoretical approaches.

#### Predict both RTs and ERs based on explicit mechanisms

Importantly, unlike all previous theories of simple visual search, the present EPIC models account extremely well for both empirical RT and ER data. They do so in ways that differ considerably from well-known prior explanations of speed–accuracy trade-offs in human performance, such as stochastic models in which information accumulation is treated as a discrete random walk or continuous diffusion process (e.g., Ratcliff et al., 2015). Those models can account for the statistics of speed–accuracy trade-offs, but they do not describe what detailed mechanisms underlie overall task performance.

In contrast, the present EPIC models overcome this deficiency. Rather than involving an abstract stochastic process, their search process relies on computational mechanisms of a general-purpose cognitive architecture. For simple visual search, the accumulated information consists of perceived visual properties of displayed objects. These properties accumulate in the architecture’s visual perceptual store as eye movements are made to inspect a stimulus display. The contents of the perceptual store are monitored by strategy rules that cause additional eye movements and, ultimately, yield a chosen manual response.

Other sources of stochasticity are also explicit in the present EPIC models. There is some simple random “noise” in the visual and motor components of the architecture. Together with the randomized display positions of stimulus objects, this contributes substantial trial-by-trial variability to predicted RTs in an otherwise deterministic system.

Nevertheless, the EPIC models yielded impressively close fits to the joint mean RT and ER data of Wolfe et al. (2010). These models also accounted well for various concomitant speed–accuracy trade-offs. Perhaps through subsequent endeavors to make such predictions, future modeling may provide additional principled accounts of conjoint factor effects on RTs and ERs in human visual search.

#### Emphasize modeling based on computational cognitive architectures

Considered overall, the present research has yielded conceptual insights, theoretical implications, and promising prospects for how to model simple visual search. As a result, this progress strongly reinforces several of our previous claims: To understand, explain, and predict high-speed human performance, the framework provided by a principled, parsimonious, empirically supported cognitive architecture like EPIC can be extremely useful and instructive.

Such architectures must be developed further and applied not only with respect to simple visual search, but also more broadly in empirical studies regarding other basic types of performance. Future research on cognitive models should take these lessons to heart and step up to a higher level of rigor, simplicity, and veracity.

## Data Availability

Modeled data were collected by other researchers and are available on cited website.

## Footnotes

1 The conventional term *feature* is ambiguous regarding a stimulus dimension (e.g. *color*) and a value on that dimension (e.g. *red*); where clarity is important, this paper uses *properties* and *values*; a *property-value* pair names a dimension and a value on that dimension.

2 Thanks are due to Susan Chipman (personal communication) for pointing this out: “Show me a model that *doesn’t* fit the data.”

## Code availability

Simulation model code has been uploaded to a repository with persistent URL (Kieras, 2025 in references)
